# Implementation of three innovative interventions in a psychiatric emergency department aimed at improving service use: a mixed-method study

**DOI:** 10.1186/s12913-020-05708-2

**Published:** 2020-09-11

**Authors:** Morgane Gabet, Guy Grenier, Zhirong Cao, Marie-Josée Fleury

**Affiliations:** 1 Management, Evaluation and Health Policies Department, School of Public Health, Université de Montréal, 7101 av. du Parc, Montreal, QC H3X1X9 Canada; 2 Research Center, Douglas Mental Health University Institute, 6875 LaSalle Blvd, Montreal, QC H4H 1R3 Canada; 3 Department of Psychiatry, McGill University, 1033, Pine Avenue West, Montreal, QC H3A 1A1 Canada

**Keywords:** Emergency department use, Brief intervention team, Crisis center team, Family-peer support team, Innovation, Implementation, Patient outcomes, Mental disorders

## Abstract

**Background:**

Emergency department (ED) use is often viewed as an indicator of health system quality. ED use for mental health (MH) reasons is increasing and costly for health systems, patients, and their families. Patients with mental disorders (MD) including substance use disorders (SUD) and suicidal behaviors are high ED users. Improving ED services for these patients and their families, and developing alternatives to ED use are thus key issues. This study aimed to: (1) describe the implementation of three innovative interventions provided by a brief intervention team, crisis center team, and family-peer support team in a Quebec psychiatric ED, including the identification of implementation barriers, and (2) evaluate the impacts of these ED innovations on MH service use and response to needs.

**Method:**

Using mixed methods with data triangulation, the implementation and impact of the three above-named ED interventions were studied. Quantitative data were collected from 101 participants (81 patients, 20 family members) using a user questionnaire and patient medical records. Qualitative data were gathered from focus groups (*n* = 3) with key intervention staff members (*n* = 14). The user questionnaire also included open-ended questions. Descriptive, comparative and content analyses were produced.

**Results:**

Key implementation issues were identified in relation to system, organizational and patient profiles, similar to results identified in most studies in the ED implementation literature aimed at improving responsiveness to patients with MD. Results were encouraging, as the innovations had a significant impact for improved patient MH service use and adequacy of care. Services also seemed adapted to patient profiles. Family members were grateful for the help received in the ED.

**Conclusions:**

Before implementing innovations, managers need to recognize the basic issues common to all new healthcare interventions: the need for staff training and strong involvement, particularly among physicians, development of collaborative tools especially in cases of potential cultural clash between staff and organizations, and continuous quality assessment. Future research needs to confirm the pertinence of these interventions, especially use of family-peer support teams in ED, as a highly innovative intervention. Broader ED strategies could also be deployed to improve MH services and decrease ED use for MH reasons.

## Background

Emergency department (ED) use is often viewed as an indicator of health system quality, especially when patients make multiple yearly ED visits [1, 2]. ED overcrowding and misuse are recurring issues internationally and in Quebec (Canada), reflecting lack of access and continuity in ambulatory care [2, 3]. From 2005 to 2017, Quebec general ED use increased by 6% [4], with 2% of this increase attributed to mental health (MH) and substance use disorders (SUD) [5]. Higher ED use exacerbates wait times and decreases care quality and patient satisfaction [6]. ED use for MH reasons including SUD [7] and suicidal behaviors [8, 9] contributes substantially to ED overcrowding [10]. Individuals with MD also have higher prevalence rates for co-occurring physical conditions, further increasing their ED use [11]. High ED use is costly and strongly indicative of inadequate care, which in turn contributes to worse patient outcomes, staff turnover and general dissatisfaction [12, 13]. For family members, caring for a loved one with MD is associated with a 29–60% probability of enduring significant psychological distress [14], which may also lead to ED use.

In this context, the need to reduce ED use while improving services and responsiveness to patient needs is crucial [2]. Innovations may be introduced within ED by MH or SUD liaison agents, for example, or developed collaboratively with outside services, e.g. crisis stabilization centers. Three basic types of innovations implemented in general or psychiatric ED, but mainly the latter, were identified in the literature [6]. The first type, “brief intervention services” (e.g. ED liaison agents, flow strategies, case management) aimed to improve ED assessment time, treatment adequacy, and appropriate patient discharge and referral to ambulatory care [15]. The second, “crisis-related services”, (e.g. crisis teams or stabilization centers, home treatment teams) help MH patients in crisis to receive more rapid and adequate help at home or through alternative services, rather than ED. Both of these innovations were developed by hospitals or community organizations working in collaboration with ED [16–18]. The third type of ED innovation, less often used, concerns “peer-user support services” established to promote patient recovery [19, 20] and focused on the reduction of psychosocial burden. These services could also be deployed for families, yet no research on family-peer support services was identified in the ED literature.

Studies have investigated the implementation of MH innovations in ED [16, 21–23], yet the evidence on their results and efficacy is mixed [15, 24, 25]. Thus, further research is needed. Concerning implementation, studies on MH innovations have investigated system-level, organizational and individual (staff, patient, family) factors [23]. Factors hindering implementation at the system level due to lack of funding, access, continuity or required intensity of services were common to the three ED intervention types [21, 22, 25], whereas organizational-level barriers related more to problems with inter-professional collaboration and communication [24, 26]. A lack of ongoing quality assessment [21] and structured referral processes [15] were key implementation factors reported in brief intervention services. The risk of cultural clash between community organization or peer support staff providing services and health care professionals, particularly concerning recognition of professional expertise, was another important issue identified [27–29]. Training and supervision of peer support staff by health professionals were viewed as key indicators of success [30]. Regarding individual factors, interpersonal skills were identified as critical [24, 26]. Certain patients were difficult to engage, such as those with personality disorders or SUD [16, 23]. Concerning the impact of ED innovations, reduction in costs [25, 31, 32], ED wait times [33–35], ED use [6, 15, 35] and hospitalization rates [16, 18, 36] were most often cited, regardless of intervention type, while the adequacy of referrals generally improved [27, 37, 38] in brief intervention and peer-user support services. In terms of health outcomes, patient symptoms decreased for the three intervention types [25, 26, 36]. Although few studies have investigated patient satisfaction [23, 39, 40] or quality of life [19], results tend to be positive.

To our knowledge, no previous study has investigated these three types of innovative ED interventions in terms of their response to patient and family needs. Moreover, the combined experiences of managers, clinicians, patients and families have rarely been investigated using survey techniques and clinical records, nor has the adequacy of care been evaluated in this context. As mentioned, studies investigating the implementation of family-peer support teams in a psychiatric ED were not identified. Improving services for families should also be a priority, given their level of burden and major role in patient MH recovery [20]. Using mixed-methods with data triangulation, this study aimed to: (1) describe the implementation of three innovative interventions provided by a brief intervention team, crisis center team, and family-peer support team in a Quebec psychiatric ED, including the identification of implementation barriers; and (2) evaluate the impact of these interventions on MH services use and response to user needs.

## Methods

### Study setting

The study was conducted in the psychiatric ED of a MH university institute in Montreal. In 2019, 3995 patients made 5980 visits to this ED, which serves the local community (368,740 inhabitants) and patients from outside areas due to its strong reputation for providing specialized MH services. MH primary care in the territory is provided by local community health service centers, general practitioners and psychologists in private practice, and by community MH organizations (e.g. crisis centers, family and patient support groups).

### Data collection

Participants were patients, family (i.e. users) or staff members providing the interventions. User members had to be at least 18 years old, and had visited the ED about 8 months prior to their inclusion in the research. Except for those served by the family-peer support team, participants had to give permission to transfer their medical records from the MH university institute to the research team. Intervention staff members referred patients or family members to researchers on a voluntary basis between January and December 2019, whereas recruitment from the family-peer support team was discontinued in May due to interruption of this service. At least 30 users for each of the three innovations were targeted for recruitment to the study, meeting minimal requirements for quantitative analyses. Recruitment was also planned for at least a one-year period to allow for the recruitment of an adequate number of users [41]. Interviews were held in a private room at the intervention offices or by telephone, and a small financial compensation was provided to patients. Staff participants were either the intervention managers or clinicians. All participants provided written informed consent. The research ethics board of the MH university institute approved the study protocol.

Data emanated from three sources: 1) a user questionnaire developed for this study, eliciting yes/no or 5-point Likert scale responses, and two open questions which users completed with interviewer assistance (supplementary file); 2) focus groups for staff involved in implementing the interventions (including a brief questionnaire for sociodemographic data); and 3) medical records for patients served by the brief intervention and crisis center teams. The user questionnaire covered: participant socio-demographic information; services received from the intervention including quality assessment; MH service use in the 12 months prior to ED visit leading to use of the brief intervention or crisis center teams, and in the 6 months after discharge from these interventions. The user questionnaire also included some clinical data not covered in medical records (i.e. perceived MH and physical health) or data that may be under-reported in medical records such as SUD, where two questions were introduced (Did you have a drinking or drug problem in the past 12 months?). For SUD data, this question and information reported in the medical records were merged. The two open questions for users concerned reasons for ED visit leading to the innovative interventions used, and an assessment of their key components. The user questionnaire required 30 min to complete. Focus groups with staff respondents used an interview guide developed for this study, which described or evaluated the following: the innovations (e.g. mandate, functioning), possible system or organizational barriers, and patient profile issues. Staff sociodemographic data were also obtained (e.g. age, sex, seniority). The focus group lasted 90 min. The user questionnaire and interview guide for the staff focus groups were developed by the research team based on the literature on innovations implemented in ED and were revised by key decision makers with strong ED expertise involved in the study.

Data from the third source, patient medical records, were collected 12 months before the designated ED visit and 6 months after discharge from the intervention, and included: patient diagnoses, illness acuity at ED visit before intervention referral, specialized outpatient services used, ED visits and hospitalization. Diagnoses were based on the International Classification of Diseases Ninth Revision (ICD-9) for outpatient services and on the Tenth Revision (ICD-10-CA) for ED visits and hospitalization. MD included: common MD (depressive disorders, anxiety disorders, adjustment disorders), serious MD (schizophrenia spectrum and other psychotic disorders, bipolar disorders), personality disorders, and SUD. Co-occurring MD and SUD, and MD and chronic physical illnesses (e.g. cancer, diabetes) were also considered (including data on SUD reported in the user questionnaire). Illness acuity was measured with the five-level Canadian Triage Acuity Scale [42], used to determine treatment priority in ED and ranging from level 1 (urgent situation with vital risk) to level 5 (least urgent).

### Analysis

This study was based on mixed methods using a sequential explanatory design [43], where qualitative findings (i.e. description of the innovation and implementation issues) are used to explain and complement the quantitative data, especially the implementation outcomes or impacts, taking into consideration patient profiles (user questionnaire). Regarding quantitative data, descriptive statistics were first produced, including percentages for categorical variables and mean values for continuous variables. Comparative analyses were conducted to test before-after differences in the interventions using chi-square tests or Fisher’s exact tests for categorical variables, and t-tests for continuous variables. The qualitative phase used a content analysis approach, where themes identified in the literature, or based on the interview guide or open questions framed the analysis, including the possibility to code emergent themes. The qualitative analysis included a four-step process [44] as follows: 1) audio-recording of interviews and verbatim transcription; 2) preliminary readings; 3) selection and definition of classification units for open-questions (patients or family members) or dimensions of the focus group interview guide (staff); and 4) separation of content into units of meaning.

Two research team members independently read the interviews with users and staff. The coding and qualitative analyses (users, staff) were produced by one team member, and validated by a second one for 30% of the content. Interrater reliability was validated at roughly 90%. To reduce the information, quantitative data (for each innovation and type of user) were summarized in tables, while the qualitative data for staff or users were summarized in a short report taking into account convergences and divergences among the innovations. This process was validated by the entire research team. Triangulation of the quantitative and qualitative data was then conducted [45], integrating user profiles (questionnaires and medical records), the three innovations including functioning and implementation issues (focus groups), services received by users, as well as other user outcomes or impacts of the innovations (questionnaire and medical records).

## Results

### Sample description (based on user or staff member questionnaires)

Of the 119 patients or family members referred by the intervention staff, 101 participated for an overall response rate of 85%: 44/48 (92%) from the brief intervention team, 37/43 (86%) from the crisis center team, and 20/28 (71%) from the family-peer support team. Average patient or family member age was 41 years old; 62% were female; 69% single and 75% had a college education (Table 1). Half worked, but only 35% full time. For 44%, family income was from Can$20,000 to $69,999. Most (81%) had close friends, three on average. Eighty-five percent of brief intervention and crisis center team participants reported a diagnosed MD: 52% common MD, 25% serious MD, 25% personality disorders, and 17% SUD; 12% reported co-occurring MD-SUD, and 2% MD-chronic physical illnesses, while 48% had experienced suicidal behaviors. Over one-third (38%) of innovation patients or family members rated their MH, and nearly a quarter (24%) their physical health, as poor or fair. Overall, crisis center intervention patients had worse living conditions than those served by the brief intervention team (e.g. fewer in autonomous housing; less education and employment; more single; more with serious MD, personality disorders, suicidal behaviours, or co-occurring MD-SUD). Response rates for intervention staff were 93%: 5/6 professionals from the brief intervention team, 5/5 from the crisis center team, and 4/4 from the family-peer support team participated in a focus group. Most staff were women (93%), 41 years old on average with 14 years of experience, 11 years of which in their current position.

**Table 1:** Patient and family member sociodemographic and clinical characteristics

	Brief intervention team		Crisis center team		Family-peer support team		Total	
	(***n*** = 44)		(***n*** = 37)		(***n*** = 20)		(***n*** = 101)	
***Sociodemographic variables***	**Mean**	**SD.**	**Mean**	**SD.**	**Mean**	**SD.**	**Mean**	**SD.**
**Age** (mean, SD.)	37.82	13.15	41.05	14.99	45.50	17.48	40.52	14.89
	**n**	**%**	**n**	**%**	**n**	**%**	**n**	**%**
**Sex**								
Female	27	61.36	24	64.86	12	60.00	63	62.38
Male	17	38.64	13	35.14	8	40.00	38	37.62
**Type of housing**								
Autonomous (house/condo/apartment)	41	93.18	27	75.00	20	100.00	88	88.00
Other	3	6.82	9	25.00	0	0.00	12	12.00
**Civil status**								
Single/separated/divorced/widowed	29	65.91	28	75.68	12	60.00	69	68.32
Married/common law	15	34.09	9	24.32	8	40.00	32	31.68
**Level of education**								
Elementary/high school	8	18.18	12	32.43	5	25.00	25	24.75
College or higher	36	81.82	25	67.57	15	75.00	76	75.25
**Work**								
Yes, full time	19	43.18	5	13.51	11	55.00	35	34.65
Yes, part time	7	15.91	6	16.22	2	10.00	15	14.85
**Family income (Can$)**								
0 to $19,999/year	10	23.26	21	58.33	2	11.76	33	34.38
$20,000 to $69,999/year	22	51.16	11	30.56	9	52.94	42	43.75
$70,000/year and higher	11	25.58	4	11.11	6	35.29	21	21.88
**Close friends**								
Yes	36	81.82	28	75.68	18	90.00	82	81.19
**Number of close friends** (mean, SD.)	3.06	1.24	3.25	1.60	3.39	1.29	3.20	1.37
***Clinical characteristics***								
**Diagnosis** ^a^								
Mental disorders (MD)	32	72.73	37	100.00	N/A	N/A	69	85.19
Common MD	21	47.73	21	56.76	N/A	N/A	42	51.85
Anxiety disorders	10	22.73	10	27.03	N/A	N/A	20	24.69
Depressive disorders	11	25.00	14	37.84	N/A	N/A	25	30.86
Serious MD	7	15.91	13	35.14	N/A	N/A	20	24.69
Schizophrenia	5	11.36	4	10.81	N/A	N/A	9	11.11
Bipolar disorders	2	4.55	9	24.32	N/A	N/A	11	13.58
Personality disorders	5	11.36	15	40.54	N/A	N/A	20	24.69
Substance use disorders ^b^ (SUD)	7	15.91	7	18.92	N/A	N/A	14	17.28
Co-occurring MD-SUD	3	6.82	7	18.92	N/A	N/A	10	12.35
Co-occurring MD-chronic physical illnesses	0	0.00	2	5.41	N/A	N/A	2	2.47
**Suicidal behaviors (ideation and attempt)** ^a^	11	25.00	28	75.68	N/A	N/A	39	48.15
**Triage priority level** ^a^					N/A	N/A		
Level 1 (immediate care)	0	0.00	0	0.00	N/A	N/A	0	0.00
Level 2–3 (urgent/very urgent care)	8	18.18	18	48.65	N/A	N/A	26	32.10
Level 4–5 (not urgent/less urgent care)	36	81.82	19	51.35	N/A	N/A	55	67.90
**Perceived mental health (MH)**								
Poor or fair	13	29.55	17	45.95	4	20.00	34	38.33
Moderately good or good	30	68.18	16	43.24	8	40.00	54	50.00
Very good or excellent	1	2.27	4	10.81	8	40.00	13	11.67
**Perceived physical health**								
Poor or fair	10	22.73	11	29.73	3	15.00	24	23.97
Moderately good or good	23	52.27	19	51.35	6	30.00	48	47.11
Very good or excellent	11	25.00	7	18.92	11	55.00	29	28.93

^a^Clinical characteristics (included diagnosis, suicidal behaviors and triage priority level): only for brief intervention and crisis center team patients (*n* = 81)

^b^These results combined information from patient clinical records and the user questionnaire (i.e. Did you have any problems with alcohol or drug consumption in the past 12 months?) We found 9 people who identified SUD from the user questionnaire only, 1 SUD from patient clinical records only, and 4 SUD from both sources

### Description of the three innovations (based on focus groups with staff members and statistics reported from the user questionnaire)

Implemented in 2016, the brief intervention team included a part-time psychiatrist (18 h/week), a full-time nurse and an administrative agent, who managed 160 patients yearly during working hours for an average of six visits per patient over 5.5 months (Table 2). The ED referred patients assessed as priority 2–3 in the triage, patients from territories not served by the MH university institute, or others without MH follow-up. All patients received brief psychosocial interventions including crisis resolution. Medication was managed for 95% of them. After discharge, 61% were referred to outpatient services: 34% to public primary care, 32% to community organizations, and 25% to specialized services.

**Table 2:** Patient and family member use of interventions and perceived quality of mental health (MH) services

	Brief intervention team (*n* = 44)		Crisis center team (*n* = 37)		Family-peer support team (*n* = 20)	
	n/mean	%/SD.	n/mean	%/SD.	n/mean	%/SD.
**Psychosocial treatment/support**	44	100.00	25	67.57	20	100.00
Number of interventions/supports (mean, SD.)	5.95	4.47	3.38	4.46	1.00	0.00
Duration of service/support in weeks or minutes (mean, SD.) ^a^	24.41	22.60	8.00	3.19	37.5	24.20
**Medication**	42	95.45	N/A	N/A	N/A	N/A
**Crisis accommodation**	N/A	N/A	29	78.38	N/A	N/A
Duration of crisis accommodation in days (mean, SD.)	N/A	N/A	6.83	3.33	N/A	N/A
**Referrals to MH services**						
Overall ^b^	27	61.36	N/A	N/A	16	80.00
To public primary care	15	34.09	N/A	N/A	N/A	N/A
To community organizations	14	31.82	N/A	N/A	16	80.00
To specialized services	11	25.00	N/A	N/A	N/A	N/A
**Overall quality of MH services** (mean, SD.)^c^	2.72	0.28	2.64	0.42	2.99	0.05

^a^Information on brief intervention and crisis center teams is provided in weeks, and information for the family-peer support team in minutes, considering that this service is offered in the emergency department (ED) during patient visit

^b^Overall references included: to public primary care (e.g. family doctors, MH services at local community health service centers), community organizations (e.g. housing, peer-user services, work support resources), and outpatient specialized services (e.g. outpatient MH clinics, day hospitals, addiction rehabilitation centers)

^c^Measured on a three-point Likert scale (1: not at all or slightly in agreement, 2: moderately in agreement, or 3: in agreement or in complete agreement), integration questions related to quality of the contact, treatments and references to services

The crisis center team, created in 1987 and reactivated in recent years, included one clinician working in the ED on a half-day/week basis, who transferred ambulatory patients to the crisis center after ED evaluation and discharge. A community organization offering short-term accommodation and community crisis follow-up, with 12 full-time professionals on staff, managed the team. About 150 ED patients were referred to the crisis center team yearly. They included patients prioritized as 4 or 5 in the ED triage, those in crisis from bereavement, separation, job loss, etc., not considered dangerous and treatment compliant. Most (78%) received three-day crisis accommodation on average, and 66% received a mean of three psychosocial interventions over an average 8-week period.

Finally, the family-peer support team started in 2017 with inclusion of a family-peer staff member in the ED in conjunction with the community organization, which included 9 full-time staff offering MH information, individual family or group support, respite services and outreach to families of patients with MD who presented directly at the ED. Flyers available in the ED provided further information about the service. The family-peer support team reached about 35–40 families monthly until May 2019 when the service terminated for lack of resources. Families of ED patients were offered psychosocial support, either single sessions (usually one only) or telephone support. Most (80%) were referred to the family-peer support group for additional follow-up. For all three interventions, service quality was rated as adequate: from 2.99 for the family-peer support team to 2.72 for the brief intervention team, based on a 5-point Likert scale.

### Factors hindering implementation of the innovative interventions (focus groups with staff members)

According to the qualitative results, patients who used the ED and one of the three interventions did so because MH services had not responded adequately to their needs for access to or continuity of services. They identified the following MH system failures: insufficient knowledge of MD among family doctors, lack of specialized MH services, unacceptably long wait times for psychiatrists or primary care psychosocial services in local community health service centers, lack of outpatient MH services or insufficient intensity in follow-up (Table 3).

**Table 3:** Box quotations

**1) Main reasons for using psychiatric emergency departments (ED) based on individual patient interviews:**	
***a) Inadequacy of mental health services in responding to their needs***	
“Family doctors are not qualified on the subject. It takes the psychiatrist. The family doctor cannot treat psychological problems!” (Brief intervention team-MB11139)^a^	
“I have the impression that doctors or other clinicians do not know what to do with someone who is suffering or that they are able to explain well what the person has. You know it’s like I’m not taken seriously.” (Brief intervention team-SCG11126)	
“I don’t think doctors or clinicians in general are qualified to handle complex cases and maybe they take things a little too personally.” (Crisis center team-AM22228)	
“At the local community health service center, when I went there to get information, the person I met was rushed. I was not lucky. I did not come across someone who had compassion and wanted to help me.” (Crisis center team- MD22202)	
“Only specialized treatment is insufficient for her.” (Family-peer support team-JJ33304)	
***b) Problems with access to mental health services***	
“Yes, apart from the fact that everything that is private is chargeable, when I approach a local community health service center and not a walk-in clinic, there was a 6 month wait just to get an evaluation. What do I do with myself during all this time? Well I end up back here at the ED. What do you want me to do?” (Brief intervention team- CV11134)	
“I tried to call three places to see, to have a psychiatrist, and then everyone passed to buck to me. I was constantly told that I was in the wrong place when I called the number the doctor had given me. It’s really badly organized, leaving patients to commit the irreparable, because you feel completely abandoned by the system. If I didn’t have the ED, I don’t know what I would have done.” (Brief intervention team-MM11114)	
“Services other than the ED are generally closed when we need them.” (Crisis center team- IG22220).	
“Sometimes services are needed quickly, and unfortunately getting access to services other than the ED can take a long time.” (Family-peer support team-CL33319)	
***c) Problems with continuity of mental health services***	
“It is difficult to get a follow-up for medication. When you start a medication, it can be difficult after that to find a doctor who is willing to continue the treatment and follow up with the patient, to maybe change the dose and accompany him along the way. It’s difficult to find.” (Brief intervention team- LG11137)	
“I had services, but the continuity has been broken.” (Crisis center team-IG22220).	
“Sometimes users need more services. When outpatient services are not enough, they are sometimes forced to go to the ED.” (Family-peer support team-CL33319)	
**2) Conditions for successful innovation according to staff (Focus groups with managers and clinicians)**	
***a) Health system:*** *Underfunding*	
“Another challenge is that we are the least funded crisis center in Montreal. We would have wanted a liaison agent, who could work a full day at the ED every week. But it isn’t possible with the staff we have now.” (Crisis center team-01)^b^	
“But I think the major weak point is money. Less than half of our budget comes from government on a stable basis. This is a major challenge.” (Family-peer support team-01)	
*Long delay to find long term follow-up in the community*	
“Unfortunately, the entire network is very bogged down, whether primary or specialized services. Thus, the delays are long before patients receive follow-up, which means that they are kept on the team longer and the caseload increases.” (Brief intervention team-01)	
*Primary care provided by two integrated university health service centres*	
“We refer people to the primary care clinic close to our ED, which is not in the same integrated health service center as us. So, we sometimes have major communication challenges. There are many different contact persons to speak with, and sometimes we encounter barriers.” (Brief intervention team-03)	
***b) Inter-organizational relationships:*** *Culture clashes*	
“Well from the outside, we had a credibility problem. We are a community organization, a non-profit organization. We were viewed as volunteers rather than professionals, whereas we have had a very professional team right from the beginning. We were a team of professionals that wanted to work in the community, but we were not always considered professional. So we faced accountability, credibility issues. We had to build trust. Because having people referred to us brings a lot of responsibility with it; we were in two completely different practice cultures.” (Crisis center team-01)	
“I think that’s probably the most serious challenge. Because it’s really about changing culture, changing mindsets; and who are we to change the hospital culture? Changing mindsets is really something that the hospital has to think about. So we do what we can, but I think there is some progress.” (Family-peer support team-01)	
***c) Organization:*** *Manager turnover*	
“In the past few years, the ED has had six different medical chiefs. So each time we certainly had to “resell” the crisis center team, reestablish links, recreate the partnership. Because a lot depends on individual will at the ED, the people with whom we need to chat, collaborate, address difficulties. So that kind of change can take us a few steps back rather than advancing.” (Crisis center team-01)	
*Problems of office space*	
“Access to offices, how to get that at the ED? It has always been complicated. We don’t have a key or an access card. We can’t move around. This is still an issue today.” (Crisis center team-01)	
“We have a lack of space, and it’s not just me! I sometimes don’t have an office where I can meet families.” (Family-peer support team-04)	
*Delay before patients receive their ED discharge*	
**“**We get stuck in the ED discharge process, because we often have to wait to receive “the go ahead” from the ED psychiatrist who authorizes patient discharges, which allows us to transfer patients to the crisis center.” (Crisis center team-02)	
***d) Clinician:*** *Turnover*	
“While some psychiatrists helped us a lot to move forward, as soon as they left things fell back! There are good practices that we had started to implement, but then slacked off.” (Crisis center team-03)	
“There are 15 different psychiatrists working in the ED, all of them part-time, and turnover is high.” (Crisis center team-04)	
*Absence of interest or knowledge*	
“Whenever Dr. M… tried to meet with other ED doctors and explain to them what the brief intervention team was, the doctors did not show up. We tried several times, and there were only one or two doctors, and always the same ones who came; the others didn’t. So, the ED doctors aren’t thus very aware of what brief intervention entails.” (Brief intervention team-02)	
***e) Patients:*** *Profiles more difficult to follow-up*	
“Co-occurring disorders are incredibly prevalent! Especially those involving use of substances like amphetamines. This contributes to the high incidence of individuals with suicidal ideation who end up at the ED; this just cannot be! Or alcoholics as well: people with alcohol problems...” (Brief intervention team-04)	
“People with autism spectrum disorder are complex to treat and refer to outpatient services.” (Brief intervention team-05)	
“As for people in the justice system for serious crimes, we are not really equipped to treat them, I would say.” (Crisis center team-02)	
“Also those with aggressive behaviors, it’s difficult to get their cooperation.” (Crisis center team-04)	
“People with antisocial personality disorders are very difficult to deal with.” (Crisis center team-01)	
“Whether psychotic or not, the person who is very, very distrustful, who has a paranoid profile, poses a challenge. We have many clients with this profile. For example, we have some who only talk about people following them on the street, or neighbors who persecute them, and we can’t talk about anything else, so that’s the problem.” (Crisis center team-01)	
*Patients estranged from their families*	
“Because there are ED users who are admitted with police escort or things like this. Sometimes the families are contacted a week or two after patients are admitted. Sometimes patients have been missing for months; they no longer have family contact.” (Family-peer support team-03)	
*Patients who do not allow ED professionals to contact their families*	
“Let’s say that a patient comes to the ED based on a psychiatric assessment order, and then systematically refuses to allow us to contact their family. We don’t necessarily have access to the family right away.” (Family-peer support team-04)	
*Aggressive or overly critical family members*	
There are sometimes families that I am not comfortable referring to the family-peer support team, because they are too aggressive. There are also at times families who, unfortunately, have behaved inappropriately in their contacts with the ED. In those cases, I would not be comfortable leaving them alone with the family-peer support team. For example a man who shouts at everyone; then I send this man out for a walk. I can understand that the man is probably in distress, but the fact remains that I don’t want to put the family-peer support team in this situation.” (Family-peer support team-03)	
**3) Most valued intervention features according to patients or family members (Individuals interviews)**^a^	
***a) Quality of the contact***	
“I appreciated the fact that the doctor listened to me. He took the time to understand what I was saying and managed to read between the lines.” (Brief intervention team-CV11134)^a^	
“Their welcome, the human approach, their friendliness and compassion, their honesty, the fact that they put us at ease in the center. They come to the ED to see us. Once at the crisis center, we meet with them at least once a day. This service was great; it went well for me. I was stressed. It helped me a lot to see clearly how to deal with problems in my life. The center was very organized; they had accommodation rules. The people who stayed with me were respectful. Overall, I had a great experience.” (Crisis center team-CG22226)	
“The crisis center where we can get a short period of respite: it’s a warm house; it’s surrounded by trees.” (Crisis center team- VS22230)	
“People help each other. People love each other. They want to help others. It’s an incredible feeling. That’s why I love the place.” (Crisis center team- VN22226)	
“I found the representative from the family-peer support team very sensitive, listening. I felt that they really wanted to help me.” (Family-peer support team-LC33306)	
***b) Quality of treatment***	
“I appreciated the availability, the professionalism of the team, the fact that I was not left in the dark about medication changes. Information about the medication and side effects was clearly explained to me. They also explained the other steps that could be followed to get better.” (Brief intervention team-MG11123)	
“We offer comprehensive psychosocial support that goes beyond medication management only.” (Crisis center team- AS22203)	
“They made sure I got the information. They made sure the information was useful to me. This was the part I liked the most.” (Family-peer support team-KM33313)	
***c) Access to care***	
“What I appreciated was that I could see a clinician quickly, and that I could call them when I had important problems that stressed me. They listened, and helped me to overcome my problems.” (Brief intervention team-EG11132)	
“It’s a welcoming environment where you can get support even during the night, which I didn’t use, but it was possible.” (Crisis center team-AS22203)	
“I really appreciated the availability of the phone service 24 h a day.” (Crisis center team- MD22202)	
“I appreciated that the help was immediate when I spoke to them.” (Family-peer support team- KM33313)	
***d) Continuity of care***	
“They called me often to remind me that I had an appointment, to see how I was doing with the medication.” (Brief intervention team-MB11140)	
“I really appreciated that they were following up people like me on a daily basis. I really enjoyed being able to speak with someone on the team every day.” (Crisis center team- LM22237)	
“When I called them, and they had to call me back, and when they told me they would send me resources, they did. They were quick. They followed me well.” (Family-peer support team- KM33313)	

^a^This code corresponds to an allocated abbreviated name for each user (related to the research questionnaire), the targeted innovation (111 = brief intervention team, 222 = crisis center team, 333 = family-peer support team) and the order in which the participant was recruited (the last two numbers)

^b^This code corresponds to the number ascribed to each staff participant (managers and clinicians) recruited through the focus groups

According to staff, system-level barriers included underfunding for MH, especially ambulatory care, and community follow-up, which forced teams, particularly the brief intervention team, to support patients longer than expected. Community organizations such as the crisis center and family-peer support teams were disproportionately underfunded, which limited potential ED partnerships, especially for the crisis center, and contributed to the termination of the family-peer support innovation.

Organizational challenges affected inter-organizational collaboration, frequent staff turnover, engagement of psychiatrists, operations and space, and culture clash. The MH university institute covered territories that operated differently, while the brief intervention team had to refer patients to providers in these diverse localities, which involved important challenges. Staff turnover particularly affected ED managers and physicians. Physicians were numerous, as most work in the ED part time, yet this hindered their involvement with the new interventions. Physicians also seemed especially uninterested, with very few attending information or management sessions on the innovations. Operational and space challenges mainly affected the crisis center and family-peer support teams. Crisis center staff had to arrange for patient transfers from the ED, often involving long waits for patient discharge. Few confidential and quiet spaces were available in the ED to the crisis center and family-peer support teams for patient or family member follow-up and psychosocial support. As they represented community organizations, ED professionals tended not to recognize sufficiently the expertise of either teams, which reduced the number of patient referrals to the crisis center. As for the family-peer support team, the integration of family perspectives and coordination with ED staff were not viewed as optimal.

Concerning patient profiles, those with antisocial personality disorders and problems with the law were evaluated as difficult to treat, as were family members who became aggressive or overly critical of peer treatment. Some ED patients also forbade clinicians from contacting their families about their conditions or from integrating family into their treatment. Others had little contact with their family.

### Innovation impact

Based on the qualitative investigation included in the user questionnaire, patients and family members mainly underlined that staff were compassionate and sensitive to them, that they listened carefully and genuinely helped. Patients also reported receiving rapid treatment; the treatment steps were explained, subsequent appointments set, and information on MH services made available. In the brief intervention team especially, medication effects were adequately explained, and comprehensive treatments offered, including psychosocial interventions. Patients especially appreciated their short respite periods in the comfortable and friendly environment of the crisis center, as well as the ability to reach clinicians on a 24/7 basis. As with the family-peer support team, crisis services were much appreciated for their easy access from the ED and the possibility to hold consultations during the wait period at the ED (Table 3).

As reported in patient medical records or the user questionnaire, overall MH service use diminished significantly among patients after discharge from the brief intervention and crisis center teams: 57% of participants used MH services post-discharge compared with 72% prior (Table 4). Regarding specific services, 69% of patients consulted general practitioners before using the interventions, compared with 41% afterward; six outpatient hospital services were provided before and four after the use of the interventions; 42% of patients used the ED before vs 25% after discharge, and 35% were hospitalized before vs 19% after discharge. Crisis center team patients were followed-up in MH services more often than patients with the brief intervention team. In fact, for the 6-month period after discharge from the crisis center team, no significant decrease was observed in overall service use for this specific group (76% before vs 70% after, compared with 68% vs 45% for the brief intervention team). However, a significant reduction in outpatient hospital service use (7 before vs 5 after) and hospitalization (52% vs 30%) were recorded. Regarding adequacy of care, patients, particularly those with the brief intervention team, claimed that MH services moderately addressed their needs, with a significant positive change after discharged from the interventions as compared with before (2.65 vs 2.22 overall, and 2.70 vs 2.05 for the brief intervention team). Regarding reasons why needs were not met, 89% of patients identified system problems (e.g. accessibility, quality of services), 59% individual reasons (e.g. no time, stigmatization), and 18% preferred to manage on their own.

**Table 4:** Mental health (MH) service use

	Brief intervention team (***n*** = 44)				Crisis center team (***n*** = 37)				Total (***n*** = 81)			
	Before ^a^		After ^b^		Before ^a^		After ^b^		Before ^a^	After ^b^		
	n/mean	%/SD.	n/mean	%/SD.	n/mean	%/SD.	n/mean	%/SD.	n/mean	%/SD.	n/mean	%/SD.
Has a family doctor	31/41	75.61	N/A	N/A	31/34	91.18	N/A	N/A	62/75	82.67	N/A	N/A
***Use of services***	30	68.18	20^1^	45.45*	28	75.68	26^1^	70.27	58	71.60	46^1^	56.79*
**Primary care**	(*n* = 30)		(n = 20)		(*n* = 28)		(*n* = 26)		(*n* = 58)		(*n* = 46)	
General practitioners	20	66.67	5^1^	25.00**	20	71.43	14^1^	53.85	40^1^	68.97	19^1^	41.30**
Local community health service centers	12	40.00	9^1^	45.00	10	35.71	7^1^	26.92	22^1^	37.93	16^1^	34.78
Psychologists (private practice)	12	40.00	8^1^	40.00	12	42.86	8^1^	30.77	24^1^	41.38	16^1^	34.78
Community organizations	5	16.67	2^2^	10.00	12	42.86	7^1^	26.92	17^1^	29.31	9^1^	19.57
**Specialized services**												
Outpatient psychiatrists	5/30	16.67	0/20^2^	0.00	19/28	67.86	14/26^1^	53.85	24/58	41.38	14/45^1^	31.11
Outpatient hospital services	2	4.55	2^2^	4.55	24	64.86	25^1^	67.57	26	32.10	27^1^	33.33
Number of different types of outpatient hospital services (mean, SD.) ^c^	1.00	0.00	1.00^3^	0.00	1.38	0.65	1.28^3^	0.61	1.35	0.63	1.26^3^	0.59
Frequency of overall outpatient hospital services used (mean, SD.)	1.00	0.00	2.00^3^	0.00	6.79	7.20	4.80^3^	3.28***	6.35	7.09	4.59^3^	3.24***
Duration of overall outpatient hospital services used in weeks (mean, SD.)	14.64	14.45	5.14^3^	1.62	31.70	16.30	15.67^3^	7.59	30.38	16.56	14.89^3^	7.82
Emergency department (ED) visits	14	31.82	6^1^	16.22*	20	45.45	14^1^	37.84	34	41.98	20^1^	24.69*
Number of ED visits (mean, SD.) ^d^	1.43	0.85	1.83^3^	0.75	2.20	1.58	1.79^3^	0.89	1.88	1.37	1.80^3^	0.83
Hospitalization	5	11.36	4^2^	10.81	23	52.27	11^1^	29.73**	28	34.57	15^1^	18.52*
Number of hospitalizations (mean, SD.)	1.20	0.45	1.25^3^	0.50	1.61	0.89	1.09^3^	0.30	1.54	0.84	1.13^3^	0.35
**Adequacy of MH services** (mean SD.) ^e^	2.05	0.82	2.70^3^	0.47**	2.42	0.73	2.62^3^	0.64	2.22	0.80	2.65^3^	0.57**
**Reasons why needs were not met** (Denominator: n)	(*n* = 28)				(*n* = 16)				(*n* = 44)			
Preferred to manage on his/her own	2	7.14	N/A	N/A	6	37.50	N/A	N/A	8	18.18	N/A	N/A
Reasons related to healthcare system ^f^	24	85.71	N/A	N/A	15	93.75	N/A	N/A	39	88.64	N/A	N/A
Reasons related to the person (individual reasons) ^g^	14	50	N/A	N/A	12	75	N/A	N/A	26	59.09	N/A	N/A

^a^Measured at 12 months before ED visit with referral to an intervention

^b^Measured at 6 months after discharge from the intervention

^c^Types of outpatient services used including brief evaluation and intervention programs, outpatient clinics (specialized MD), day hospitals, and other types of specialized outpatient MH services (e.g. electrophysiology, nutrition)

^d^This account of the 12-month ED visits did not include the ED visit leading to the interventions

^e^Measured on a five-point Likert scale (1: the services are not adequate to 5: the services are entirely adequate)

^f^Healthcare system reasons included: help isn’t readily available, language problems, don’t have the financial means, insurance doesn’t cover the costs, don’t have confidence in the services, dissatisfied with the quality of services

^g^Individual reasons included: don’t know how or where to obtain the type of help appropriate to my problem, don’t find time to look after it, employment or occupation prevented me from seeking help, afraid of what others will think of me, prefer to count on my family or friends to help me

^1,2,3^Comparison analyses were conducted to assess statistical differences between groups (before and after) by teams (brief intervention team, crisis center team and total): ^1^ Chi-square test or ^2^ Fisher’s exact test were used for categorical variables and ^3^ t-tests for continuous variables. Significance indicated by: *p* < 0.001***; *p* < 0.01**; *p* < 0.05*

## Discussion

This study aimed to describe the implementation and impact of three innovative interventions designed to improve service use in a Quebec psychiatric ED and better respond to the needs of patients and family members. The three interventions examined in this study differed in certain respects from interventions described in the literature. As a new type of innovation, the brief intervention team was similar to the brief case management strategy, identified in the literature, in providing both medication and psychosocial interventions [46]. This innovation also resembled the flow strategy [15], and liaison psychiatry services implemented mainly in general ED [33], given the importance of appropriate referrals to community MH service providers for this clientele [23]. However, these interventions served patients without current MH follow-up and those residing outside the hospital territory, and not only patients with co-occurring MD-SUD or specific physical illnesses as found in the literature for the other related interventions. Rather, the team described in this study served a clientele without family doctors, helping them to avoid inappropriate ED use. The crisis center and family-peer support teams, as community services that collaborated closely with ED staff, met the recommendations of the American Psychiatric Association for potentially reducing ED and hospitalization [27] by offering viable alternatives [16, 47]. The major difference between the crisis center team intervention in this study and home treatments teams described in the literature was that treatment was provided in a small community organization, which offered a homelike, friendly environment [17]. Moreover, clients of the crisis center team were experiencing psychosocial crisis more than MH crisis, as envisaged in the original model [18]. While peer users in ED were the subject of a clinical demonstration project [48], the inclusion of a family-peer in the ED has not been previously reported as already mentioned. The family-peer represents an interesting strategy for reaching families and offering them coping tools to better support loved ones with MH issues and their own wellbeing, a strong advantage that was reported by all group study members interviewed. Family-peer staff played the same role as peer-user staff at the ED or in similar health teams, counseling peers through their own MH experiences [20].

Regarding factors that hindered implementation identified in this study through focus groups, most were previously reported in the ED literature and related mainly to heath system issues: lack of resources for adequately meeting patient needs, long delays in accessing psychosocial or psychiatric services, and insufficient intensity of MH follow-up [31]. ED use is often reported in the literature as resulting from lack of MH expertise among family practitioners [49–51] and was observed in this study by both users and staff members. Community services like the study crisis center and family-peer support teams were also disproportionately identified as underfunded [31], contributing to high ED use and incentivizing the brief intervention team to support patients longer than planned. In the MH university institute where the study took place, roughly half of ED patients were prioritized as 4 or 5 at triage [42]; that is, they were less urgent cases more appropriate for ambulatory care than the ED.

Regarding organizational issues, staff members underlined communication problems between staff and organizations, often related to their distinct cultures, values and practices as observed in other studies [25, 27, 28]. While recovery is now central to MH treatment, the lack of appreciation for peer expertise among health professionals reported in our study is frequently mentioned in the literature [19, 20], as is the problem of staff turnover [52–54] and insufficient involvement in innovations [23], all important barriers to implementation also identified in this study. Lack of private space in the ED for patient or family member consultations, reported in the qualitative findings, was previously identified in a study of brief intervention services [23]. Concerning individual barriers, difficulties in treating patients identified by staff participants were like those identified in previous studies as affected by stigma, e.g. patients with co-occurring MD-SUD or personality disorders. MH professionals in the ED, like those in primary care, are often considered reluctant to treat patients with more serious or complex MD [55]. Finally, the desire of families to be engaged in the care of their loved ones was strongly hindered by patient confidentiality issues according to our findings, as in previous research [56].

Overall, patients and family members in this study appreciated the three innovative ED interventions, as in related studies [16, 23, 39]. We found that empathy and active listening skills among staff, including peers, had direct benefit for patients or family members, as previously reported [39]. Studies have also emphasized that good patient-carer therapeutic relationships improve care continuity [57, 58] and patient functioning [46]. These aspects were also confirmed by staff members who identified these qualities as facilitating factors for these types of innovation. In this study, patients received timely treatment and follow-up by innovation staff following ED discharge, contributing to patient satisfaction. Research shows that the shorter the wait time the more patients were satisfied with services [59, 60]. Biopsychosocial treatments including medication management were previously found to increase patient satisfaction [46], as also evidenced in the present brief intervention team. Surveys have found that patients in crisis prefer to be treated in a friendly, homelike environment like that offered in the study crisis center team rather than in ED or hospitals [30, 36].

The reduction in patient service use after discharge from these innovative services coincided with previous findings [6, 15, 18]. ED use diminished particularly among patients with the brief intervention team, similar to results in liaison psychiatry services [33], flow strategy [15] and brief case management studies [61]. Consultations with general practitioners also significantly diminished and perceived adequacy of care increased. To our knowledge, these results have not been previously reported, suggesting that the frequency and type of help received responded adequately to patient needs and improved their MH conditions. Studies on innovations similar to those of the brief intervention team have reported decreased symptoms [26], suicidal behaviors [22, 34] and psychosocial problems [46]. Since most patients (61%) were given MH referrals following the brief intervention, it may be hypothesized that these referrals were adequate and that MH network functioning was effective [62, 63]. However, an alternative explanation for this decrease in service use may be that patients never accessed services, that discontinuity in care occurred or that patient non-adherence to services increased after discharge from the intervention and referral – however the study found that patients were generally satisfied with services. As with use of the crisis center team in this study, a significant reduction in use of outpatient hospital services and in hospitalization after discharge have been reported [18, 35]. These results corresponded to the profiles of patients with more serious MH using in the study more the crisis center, who usually are also treated in specialized services and experience frequent hospitalization [18, 47]. Research on adequacy of help among patients confirms the difficulty of treating patients with more serious and complex MH profiles, as in the present group, and in meeting their needs [16]. Thus, they often require specialized, ongoing, and intensive outpatient care.

### Limitations

This study had certain limitations. First, as the sample was selected from a psychiatric ED in a Quebec MH university institute, and on a voluntary basis, the findings may not be generalizable to all ED, and particularly not general ED or those located in semi-urban or rural areas. Second, the innovations targeted few ED patients compared with the overall yearly patient volume in the hospital ED. More effort is needed to promote and improve ED efficiency and response to patients with MH needs and their family members. Also, the small sample size for users of each ED intervention prevented the generation of more complex statistics, especially for the family-peer user group. Third, it was also difficult to assess the family-peer support team, as no research on this type of intervention was identified in the literature. Finally, the six-month assessment period for investigating outcomes may be insufficient to ensure that results are sustained.

## Conclusion

This study was original in focusing on brief interventions in a Quebec psychiatric ED for a clientele without family doctors, and on crisis prevention and the use of family-peer support teams for improving MH services and for reducing ED use and hospitalization among MH patients. Implementation issues identified in this study were similar to those reported elsewhere in the context of ED or in the general implementation literature. Managers need to better identify key issues before embarking on implementation initiatives: improving staff training and actively involving them in new interventions, particularly physicians; developing collaborative tools geared especially to preventing cultural clashes between staff and organization, and encouraging continuous quality assessment. Reduced patient service use after discharge reflected the positive impact of these innovative interventions, leading to greater adequacy of care for patients as well as improved use of outpatient MH services, which seemed adapted to patient profiles. Family members were grateful for the help they received in the ED, even though this type of intervention would need to be more broadly investigated. Future research needs to confirm the pertinence of these interventions and promote broader ED strategies to improve access to MH services and decrease ED use for MH reasons.

## Supplementary information

**Additional file 1.**

## Data Availability

In accordance with the applicable ethics regulations in the province of Quebec, the informed consent form must specifically inform the participants of the possibility of research data sharing with third parties, as well as the limitations and safety measures associated with such data sharing. Since the consent form approved by the Douglas Mental Health University Institute Research Ethics Board signed by participants for this research project did not specifically provide for such data sharing, the principal investigator is responsible towards the participants to keep these data confidential.

## Abbreviations

ED: Emergency Department
MH: Mental health
SUD: Substance-use disorders
MD: Mental disorders
ICD: International classification of diseases
