# Supplementary material for: Implementation of three innovative interventions in a psychiatric emergency department aimed at improving service use: a mixed-method study
Source: BMC Health Serv Res. 2020 Sep 11;20:854. doi: 10.1186/s12913-020-05708-2 (PMC7488576; doi:10.1186/s12913-020-05708-2)
Supplement: Supplementary file 1 — Additional file 1. [file 12913_2020_5708_MOESM1_ESM.docx]

**Questionnaire: for Service users at La Relance on the quality of support offered and service coordination**

Interview date:

Service user code:

Interviewer name:

**Introduction**

You used the Douglas Emergency Department during the **past 3 months**, and were referred to services at la Relance. I am going to ask you some questions about your visit to the emergency, your utilization of mental health services, other services at la Relance, and your current support. These questions will help us to understand your experience and your satisfaction with the services you have received, in order for us to make recommendations aimed at improving mental health services.

**A. OVERVIEW OF EMERGENCY SERVICES USE**

**1. In a few words, for what reason(s) did you go to the Douglas emergency, at the time of your referral to the services of La Relance? (Interviewer: specify the date given by la Relance)**

___________________________________

****Interviewer: record the patient’s response, and also summarize the response in writing on the server of your tablet.***

**2. In the past 12 months and including this visit on ______________ (interviewer: specify date), how many times did you come to the Douglas Emergency for mental health reasons or for consumption?**

**□** 1=1

**□** 2=2

**□** 3=3

**□** 4=4

**□** 5=5

**□6= Other**

**2.1 *Specify the number of times, if other:***________

**3. In the past 12 months, did you go to any other emergency than the Douglas emergency for mental health reasons?**

**1= Yes**

**0= No** *(Skip to question 4)*

**3.1 If yes, how many times did you visit emergency departments, excluding the Douglas, in the past 12 months?**

**□** 0=0

**□** 1=1

**□** 2=2

**□** 3= 3

**□** 4= 4

**□** 5= 5

**□** 6= Other (If Q3.1 is less than or equal to 5, skip to question 4)

***3.2.If other, specify the number of times:_________________***

**4. In the past 12 months, and before using services at the Douglas emergency and at la Relance (date), did you use other services for your problems related to mental health, alcohol or drug use, or problems with your emotions?**

🞅1= Yes

🞅0= No (Skip to Q4.2)

🞅99= No answer (Skip to Q4.2)

**I am going to present different types of services and ask you to indicate whether you have used them for your mental health problems, alcohol or drug problems, or problems with your emotions before using the Douglas emergency and la Relance.**

***4.1 Have you been hospitalized (sent to a ward) for mental health problems, alcohol or drug problems, or problems with your emotions?***

🞅1= Yes

🞅0= No (Skip to 4.2)

🞅99= No answer (Skip to 4.2)

***4.1.1 How many times?***

____________(specify)

***4.1.2 How satisfied were you with this service?***

🞅1= Not at all satisfied or dissatisfied

🞅2= Moderately satisfied

🞅3= Satisfied or completely satisfied

🞅99= No answer

***4.2 Do you have a family doctor?***

🞅1= Yes

🞅0= No (Skip to 4.2.2)

🞅99= No answer (Skip to 4.2.2)

***4.2.1 Have you seen your family doctor?***

🞅1= Yes

🞅0= No (Skip to 4.3)

🞅99= No answer

***4.2.1.1 How many times have you seen your family doctor?***

***_____________(specify number)***

***4.2.1.2 How satisfied were you with your family doctor?***

🞅1= Not at all satisfied or dissatisfied

🞅2= Moderately satisfied

🞅3= Satisfied or completely satisfied

🞅99= No answer

***4.2.2 Are you registered on a wait list for access to a family doctor?***

🞅1= Yes

🞅 0= No

🞅99= No answer

**4.3 *Have you had a consultation in a walk-in medical clinic with a doctor other than your family doctor?***

🞅1= Yes

🞅 0= No (Skip to 4.4.)

🞅99= No answer (Skip to 4.4)

***4.3.1 How many times have you consulted one or more psychiatrists other than at the emergency or during a hospitalization?***

____________(specify number)

***4.3.2 How satisfied were you with this service?***

🞅1= Not at all satisfied, or dissatisfied

🞅2= Moderately satisfied

🞅3= Satisfied or completely satisfied

🞅99= No answer

***4.4 Have you consulted one or more psychiatrists outside of the emergency or a hospitalization?***

🞅1= Yes

🞅 0= No (Skip to 4.5)

🞅99= No answer (Skip to 4.5)

***4.4.1 How many consultations did you have?***

**____________**(specify)

***4.4.2 How satisfied were you with this/these professional(s)?***

🞅1= Not at all satisfied, or dissatisfied

🞅2= Moderately satisfied

🞅3= Satisfied or completely satisfied

🞅99= No answer

**4.5 *Have you consulted the psychosocial team or specialized mental health team at a CLSC?***

🞅1= Yes

🞅 0= No (Skip to 4.5)

🞅99= No answer (Skip to 4.5)

***4.5.1 How many times?***

_______(specify)

***4.5.2 How satisfied were you with this service?***

🞅1= Not at all satisfied, or dissatisfied

🞅2= Moderately satisfied

🞅3= Satisfied or completely satisfied

🞅99= No answer

**4.6 *Have you seen one or more psychologists in a private practice?***

🞅1= Yes

🞅 0= No (Skip to 4.7)

🞅99= No answer (Skip to 4.7)

***4.6.1 How many times?***

_______(specify)

***4.6.2 How satisfied were you with this/these psychologists?***

🞅1= Not at all satisfied, or dissatisfied

🞅2= Moderately satisfied

🞅3= Satisfied or completely satisfied

🞅99= No answer

**4.7 *Have you received mental health, drug or alcohol, or other services from a community organization (e.g. crisis center, support services, counseling services, suicide prevention, addiction services)?***

🞅1= Yes

🞅 0= No (Skip to 4.8)

🞅99= No answer (Skip to 4.8)

***4.7.1 How many times?***

_______(specify)

***4.7.2 How satisfied were you with this service?***

🞅1= Not at all satisfied, or dissatisfied

🞅2= Moderately satisfied

🞅3= Satisfied or completely satisfied

🞅99= No answer

**4.8 *Have you received other services?***

🞅1= Yes

🞅 0= No (Skip to 5)

🞅99= No answer (Skip to 5)

***4.8.1 Which ones?***

***____________________________________*** (specify)

***4.8.1.1 How many times?***

_______(specify)

***4.8.1.2 How satisfied were you with this/these service(s)?***

🞅1= Not at all satisfied, or dissatisfied

🞅2= Moderately satisfied

🞅3= Satisfied or completely satisfied

🞅99= No answer

**4.9 *Have you received other services?***

🞅1= Yes

🞅 0= No (Skip to 5)

🞅99= No answer (Skip to 5)

***4.9.1 Which?*** (specify)

***_____________________________________________***

***4.9.1.1 How many times?***

_______(specify)

***4.9.1.2 How satisfied were you with this/these service(s)?***

🞅1= Not at all satisfied, or dissatisfied

🞅2= Moderately satisfied

🞅3= Satisfied or completely satisfied

🞅99= No answer

**4.10 Have you received other services*?***

🞅1= Yes

🞅 0= No (Skip to 5)

🞅99= No answer (Skip to 5)

***4.10.1 Which?***

***_____________________________________________*** (specify)

***4.10.1.1 How many times?***

_______ (specify)

***4.10.1.2 How satisfied were you with this/these service(s)?***

🞅1= Not at all satisfied, or dissatisfied

🞅2= Moderately satisfied

🞅3= Satisfied or completely satisfied

🞅99= No answer

**4.11 Have you received other services*?***

🞅1= Yes

🞅 0= No (Skip to 5)

🞅99= No answer (Skip to 5)

***4.11.1 Which ones?***

***_____________________________________________***(specify)

***4.11.1.1 How many times?***

_______(specify)

***4.11.1.2 How satisfied were you with this/these service(s)?***

🞅1= Not at all satisfied, or dissatisfied

🞅2= Moderately satisfied

🞅3= Satisfied or completely satisfied

🞅99= No answer

**5. I am going to read a sentence and ask you if you are: 1) not at all or slightly in agreement; 2) moderately in agreement; 3) in agreement or entirely in agreement, with this statement:**

***« Services outside of the emergency and La Relance meet my mental health needs. »***

🞅1= Not at all or slightly in agreement

🞅2= Moderately in agreement

🞅3= In agreement or entirely in agreement (Skip to 6)

🞅99= No answer (Skip to 6)

***5.1* Which of the following reasons explain why services outside of the emergency and La Relance do not meet or slightly meet, moderately meet, or entirely meet your mental health needs. Respond with a “yes” or “no” to the statements that I’m going to read:**

🞅1= You prefer to manage on your own

🞅2= You don`t know how, or where, to obtain the type of help appropriate to your problem

🞅3= You don`t find time to look after it (e.g. too busy)

🞅4= Your employment or occupation prevent you (e.g. workload, work schedule or lack of cooperation from the supervisor)

🞅5= The help isn`t readily available

🞅6= You don’t have confidence in the services.

🞅7= You don`t have the financial means.

🞅8= Your insurance doesn`t cover the costs.

🞅9= You are afraid of what others will think of you.

🞅10= There is a language problem.

🞅11= You prefer to count on your family or friends to help you.

🞅12 = You are dissatisfied with the quality of services.

🞅13= Other (Skip to 5.1.1)

🞅99= No answer

***5.1.1 Specify any other reasons for which services outside of the emergency and La Relance do not meet, moderately meet, or entirely meet your mental health needs?***

***­­­­­­­­­­­­­­­­­­­__________________________________________________***

****Interviewer: Record the response here and on the server of your tablet.***

**6. How would you rate your general knowledge of mental health or addiction services?**

🞅1= Poor or very little knowledge

🞅 2= Moderate or good

🞅 3= Very good or excellent

🞅99= No answer

**B. QUALITY OF SERVICES RECEIVED AT THE EMERGENCY**

**7. Thinking of the Douglas Emergency, please indicate for each of the following statements, whether you are: 1= not at all or slightly in agreement; 2= moderately in agreement; 3= in agreement or entirely in agreement:**

|  | **1** | **2** | **3** | **97**  **Don’t know** | **99**  **No answer** |
| --- | --- | --- | --- | --- | --- |
| Service providers at the Douglas Emergency are respectful. |  |  |  |  |  |
| Service providers at the Douglas Emergency listen to my problems. |  |  |  |  |  |
| The information received at the Douglas emergency regarding my problems and treatments is sufficient. |  |  |  |  |  |
| I was adequately informed at the Douglas Emergency about the services available in my community that may address my needs. |  |  |  |  |  |
| My visits to the Douglas Emergency did not allow me to adequately discuss my problems. |  |  |  |  |  |

**8. Thinking of services received at the Douglas Emergency, would you say that you are:**

🞅1= Not at all or slightly satisfied

🞅2= Moderately satisfied

🞅3= Satisfied or completely satisfied

🞅99= No answer

**9. Thinking back to the way in which you were referred to La Relance, would you say for each of the following statements that you are: 1) not at all or slightly in agreement; 2) moderately in agreement; or 3) in agreement or completely in agreement?**

|  | **1** | **2** | **3** | **99**  **No answer** |
| --- | --- | --- | --- | --- |
| A service provider at the Douglas Emergency explained to me the reasons for which I was referred to la Relance. |  |  |  |  |
| The information I received about La Relance was clear. |  |  |  |  |
| At the Douglas Emergency I was able to ask questions about the crisis services offered at La Relance. |  |  |  |  |
| I find that my referral to la Relance was fair. |  |  |  |  |

**C. QUALITY OF SERVICES RECEIVED AT LA RELANCE**

**10. How many times did you meet with a service provider at la Relance?**

**□** 1=1

**□** 2=2 (Skip to 11)

**□** 3=3 (Skip to 11)

**□** 4**=** Other (Skip to 10.1)

***10.1 Specify the number of times***

________

**11. With what frequency were these meetings held?**

🞅1= in the same week

🞅2= every two weeks

🞅3= Every three weeks

🞅4= Once a month

🞅5= Other (skip to 11.1)

🞅99= No answer

**11.1 With what frequency were these meetings held, if other?**

__________________________ (specify)

**12. What was the duration of the meetings at Relance, on average?**

**_____minutes _____hours**

**13. What kinds of support did you receive at la Relance? *Respond with a “yes” or “no” to the following statements that I am going to read to you*:**

🞅1= Psychological support (e.g. I was listened to.)

🞅2= Therapeutic support (e.g. They gave me advice or ways of doing things that made me feel better, and helped me better manage my mental health problems)

🞅3= Crisis support (e.g. They helped me resolve my crisis situation.)

🞅4= They gave me medication, or modified my medication.)

🞅5= They referred me to another service at the Douglas.

🞅6= They referred me to the one-stop service so that I could access a family doctor.

🞅7= They referred me to a CLSC in my neighborhood, to psychosocial services or mental health services.

🞅8= They referred me to a community organization (e.g. crisis center, support group)

🞅9= They referred me to an addiction rehabilitation centre.

🞅10= They referred me to organizations that helped me find adequate housing.

🞅11= Other (Skip to 13.1)

🞅99= No answer

*13.1 What other types of support have you received at la Relance?*

**_______________________________________**

**14. Thinking of La Relance, would you say that you are: 1) not at all or slightly in agreement; 2) moderately in agreement; or 3) in agreement or complete agreement with each of the following statements:**

|  | **1** | **2** | **3** | **97**  **Don’t know** | **98**  **Does not apply** | **99**  **No answer** |
| --- | --- | --- | --- | --- | --- | --- |
| Service providers at la Relance are respectful. |  |  |  |  |  |  |
| Service providers at la Relance listen to my problems. |  |  |  |  |  |  |
| The information I received at la Relance regarding my problems and treatments was sufficient. |  |  |  |  |  |  |
| My meetings with la Relance have allowed me to treat my problems adequately. |  |  |  |  |  |  |
| At la Relance, they informed me adequately on the services available in the community that respond to my needs. |  |  |  |  |  |  |
| La Relance put me in contact with service providers or resources that will help me with my mental health problems. |  |  |  |  |  |  |
| La Relance allowed me to avoid returning to the emergency for my mental health problems. |  |  |  |  |  |  |
| I can contact la Relance anytime in case of problems. |  |  |  |  |  |  |
| I would not hesitate to recommend la Relance to any of my friends or close relations in a crisis. |  |  |  |  |  |  |

**15. Thinking of services you have received from La Relance, could you tell us whether you are:**

🞅1= Not at all or slightly satisfied

🞅2= Moderately satisfied

🞅3= Satisfied or completely satisfied

🞅99= No answer

**16. Since your meetings at la Relance, have you used any services other than those at la Relance or the emergency for your mental health needs?**

□ 1= Yes

□ 0= No (skip to 18)

🞅98= Not applicable (skip to 18)

🞅99= No answer (skip to 18)

**I am going to present different types of services, and ask you to indicate whether you have used them « after using La Relance ».**

***16.1 Your family doctor (if 4.2=1 Yes)***

□ 1= Yes, and the referral or contact was arranged by La Relance

□ 2= Yes, but the referral or contact was not arranged by La Relance

□ 0= No, I have not used this service

🞅98= Not applicable

🞅99= No answer

***16.2 Another general practitioner at a walk-in medical clinic***

□ 1= Yes, and the referral or contact was arranged by La Relance

□ 2= Yes, but the referral or contact was not arranged by La Relance

□ 0= No, I have not used this service

🞅98= Not applicable

🞅99= No answer

***16.3 Psychosocial or mental health services at a CLSC***

□ 1= Yes, and the referral or contact was arranged by La Relance

□ 2= Yes, but the referral or contact was not arranged by La Relance

□ 0= No, I have not used this service

🞅98= Not applicable

🞅99= No answer

***16.4 Psychiatric services in a hospital (follow-up with a doctor or psychiatrist)***

□ 1= Yes, and the referral or contact was arranged by La Relance

□ 2= Yes, but the referral or contact was not arranged by La Relance

□ 0= No, I have not used this service

🞅98= Not applicable

🞅99= No answer

***16.5 Services for dealing with your problems related to consumption or gambling in an addiction rehabilitation center (CRD) or other community organization for addiction or gambling***

□ 1= Yes, and the referral or contact was arranged by La Relance

□ 2= Yes, but the referral or contact was not arranged by La Relance

□ 0= No, I have not used this service

🞅98= Not applicable

🞅99= No answer

***16.6 Psychologist in private practice***

□ 1= Yes, and the referral or contact was arranged by La Relance

□ 2= Yes, but the referral or contact was not arranged by La Relance

□ 0= No, I have not used this service

🞅98= Not applicable

🞅99= No answer

***16.7 Services at a crisis center***

□ 1= Yes, and the referral or contact was arranged by La Relance

□ 2= Yes, but the referral or contact was not arranged by La Relance

□ 0= No, I have not used this service

🞅98= Not applicable

🞅99= No answer

***16.8 Mental health support services***

□ 1= Yes, and the referral or contact was arranged by La Relance

□ 2= Yes, but the referral or contact was not arranged by La Relance

□ 0= No, I have not used this service

🞅98= Not applicable

🞅99= No answer

***16.9 Other community-based services***

□ 1= Yes, and the referral or contact was arranged by La Relance

□ 2= Yes, but the referral or contact was not arranged by La Relance

□ 0= No, I have not used this service

🞅98= Not applicable

🞅99= No answer

***16.10 Other services*** (skip to 16.10.1)

□ 1= Yes, and the referral or contact was arranged by La Relance

□ 2= Yes, but the referral or contact was not arranged by La Relance

□0= No, I have not used other services (skip to 17)

🞅99= No answer (skip to 17)

*16.10.1 What was this other service?*

*_____________________________________________________* (specify)

***16.11 Other services***

□ 1= Yes, and the referral or contact was arranged by La Relance

□ 2= Yes, but the referral or contact was not arranged by La Relance

□0= No, I have not used other services (skip to 17)

🞅99= No answer (skip to 17)

*16.11.1 What was this other service?*

________________________________________________________(specify)

**17. I am going to read a statement and ask you to tell me whether you are: 1) not at all or slightly in agreement, 2) moderately in agreement; or 3) in agreement or entirely in agreement: “The services I used after my stay at the Douglas Relance responded to my mental health needs.”**

🞅1= Not at all or slightly in agreement

🞅2= Moderately in agreement

🞅3= In agreement or entirely in agreement

🞅99= No answer

**18. If you have not used services for your mental health problems, emotions, or problems related to alcohol or drugs since obtaining support from La Relance, would you have need of such services to help you?**

□ 1= Yes

□ 0= No

🞅99= No answer

**D. SOCIO-DEMOGRAPHIC AND CLINICAL DATA**

**19. How old are you? _________years**

**20. Are you?**

**□**1= a man

**□** 0= a woman

**□** 2= other (skip to 20.1)

¸

*20.1 How would you describe your gender?*

________________________________

**21. I am going to list some types of lodging; tell me in which one you are living.**

**□** 1= Residence such as a house, condo, or rental apartment

**□** 2= Supervised apartment

**□** 3= Subsidized apartment (e.g. H.L.M. or O.S.B.L)

**□** 4= Family-type residence

**□** 5= Group home

**□** 6= No fixed residence

**□ 7**= Other (skip to 21.1)

**□** 99 = No answer

*21.1 In what type of lodging are you living, if other?*

______________________________________________________

**22. What is your marital status?**

**□** 1= Single

**□** 2= Separated/ divorced/ widowed

**□** 3= Married/ common law

**□** 4= Other (skip to 22.1)

**□** 99= No answer

*22.1 What is your marital status, if other?*

_______________________________________

**23. Do you have children?**

**□** 1= Yes, and have custody

**□** 2= Yes, but not custody

**□** 0= No

**□** 99= No answer

**24. Do you have close friends on whom you can rely in case of need?**

**□** 1= Yes

□ 0= No (Skip to 25)

□ 99= No answer

**24.1 *How many close friends do you have, on whom you can rely in case of need?***

__________________(number)

**25. What is your highest level of education?**

**□** 1= Elementary school

**□** 2= High school

**□**3= CEGEP or more

**□** 4= No schooling

**□** 99= No answer

**26** **Are you currently working?**

**□** 1= Yes, full time

**□** 2= Yes, part time

**□**0= No (skip to 26.1.1)

**□**98 = Not applicable

**□**99= No answer

***26.1* If not working, what is your current situation?**

**□**1= Student

**□**2= on unemployment insurance, or the CNESST (illness insurance)

**□** 3= on unemployment insurance or social assistance

**□** 4= Retired or annuity

**□** 5= without personal income

**□** 6= Other (skip to 26.1.1)

**□** 99 = No answer

*26.1.* ***What is your current situation?***

________________________________________

**27. In your opinion, your physical health is:**

🞅 1= Poor or fair

🞅 2= moderately good or good

🞅 3= very good or excellent

🞅 99= no answer

**28. In your opinion, your mental health is:**

🞅 1= Poor or fair

🞅 2= moderately good or good

🞅 3= very good or excellent

🞅 99= no answer

**29. Have you received one or more mental health diagnoses in the past 12 months, or a previous diagnosis that persists?**

□ 1= Yes

□ 0= No (skip to 30)

□ 99= No answer (skip to 30)

**29.1 If yes, please identify which mental health diagnosis/es you have received in the past 12 months, or previous diagnoses that persist:**

__________________________

**30.** **Have you had problems with alcohol consumption in the past 12 months?**

□ 1= Yes

□ 0= No

□ 99= No answer

**31 Have you had problems with drug in the past 12 months?**

□ 1= Yes

□ 0= No

□ 99= No answer

***31.1 If yes, please indicate which type of drug(s)?***

***­­­­­­­­­­­­­­­­­­­­­____________________***

**32. Have you had gambling problems in the past 12 months?**

□ 1= Yes

□ 0= No

□ 99= No answer

**33. Have you experienced suicidal ideation in the past 12 months?**

□ 1= Yes

□ 0= No

□ 99= No answer

**34. What was your family income in the past year?**

**_________________$**

**34.1 *I am going to present some annual salary categories and ask you to tell me which one corresponds best to your family income:***

**□** 1= 0 à 9 999$/year

**□** 2= 10 000 à 19 999$/year

**□** 3= 20 000 à 29 999$/year

**□** 4= 30 000 à 39 999$/year

**□** 5= 40 000 à 49 999$/year

**□** 6= 50 000 à 59 999$/year

**□** 7= 60 000 à 69 999$/year

**□** 8= 70 000 à 79 999$/year

**□** 9= 80 000 à 89 999$/year

**□** 10= 90 000 à 99 999$/year

**□** 11= 100 000$ et plus/year

**□** 99= No answer

**E - QUALITATIVE QUESTIONS**

**35. Recalling the overall service provided by La Relance, tell me in a few words what you most appreciated.**

*****Interviewer: record the response of the patient below, and also provide a written summary on your tablet.***

**___________________________________________________________________________**

**36. Tell me what could be improved in terms of the services offered by La Relance?**

*****Interviewer: record the response of the patient below, and also provide a written summary on your tablet.***

**___________________________________________________________________________**

**37. Other than La Relance, what would most help you to improve your overall mental health problems and better pass through periods of crisis?**

****** *Interviewer: record the response of the patient below, and also provide a written summary on your tablet.***

**We thank you kindly for your participation in our project. Your collaboration is invaluable and will contribute to our recommendations aimed at better responding to your needs!**

**Questionnaire: for Service users at L’Autre Maison on the quality of support offered and service coordination**

Interview date:

Service user code:

Interviewer name:

**Introduction**

You used the Douglas Emergency Department during the **past 3 months**, and were referred to the L’Autre Maison crisis center. I am going to ask you some questions about your visit to the emergency, your utilization of mental health services, other services you have received from l’Autre Maison, as well as your current support. These questions will help us to understand your experience and your satisfaction with the services you have received, in order for us to make recommendations aimed at improving mental health services.

**A. OVERVIEW OF EMERGENCY SERVICES USE**

**1. In a few words, for what reason(s) did you go to the Douglas emergency, at the time of your referral to the services of L’Autre Maison (Interviewer: specify the date given by L’Autre Maison)?**

___________________________________

****Interviewer: record the patient’s response, and also summarize the response in writing on the server of your tablet.***

**2.1 Before having contact with a service provider from L’Autre Maison, did you meet with a physician (general practitioner or psychiatrist)?**

🞅 1= Yes

🞅 0= No

🞅 99= No answer

**2.2 Before having contact with a service provider from L’Autre Maison, did you meet with a nurse?**

🞅 1= Yes

🞅 0= No

🞅 99= No answer

**2.3 Before having contact with a service provider from L’Autre Maison, did you meet with a social worker?**

🞅 1= Yes

🞅 0= No

🞅 99= No answer

**2.4 Before having contact with a service provider from l’Autre Maison, did you meet with another professional?**

🞅 1= Yes (Skip to 2.4.1)

🞅 0= No

🞅 99= No answer

***2.4.1 What type of professional was this? (specify)***

***______________________________________________________________***

- 1. **How much time did you spend at the Emergency on ______ (date: interviewer: specify date of the ER visit) before having contact with a service provider from L’Autre Maison?**

**_______ minutes**

**______ hours**

**3.2 How much time did you spend at the Emergency on ___________ (interviewer: specify the date of the ER visit) before your departure from the Emergency for l’Autre Maison?**

**_______ minutes**

**_______ hours**

**4. During the last 12 months, and including the visit of ___________ (date: interviewer specify date), how many times did you come to the Douglas Emergency for mental health reasons or due to consumption?**

**□** 1=1

**□** 2=2

**□** 3=3

**□** 4=4

**□** 5=5

**□** 6=Other (Skip to 4.1)

**4.1 Specify the number of times, if other:_________________**

**5. Besides the Douglas Emergency, have you made visits to other emergency rooms for mental health reasons during the past 12 months? If yes, how many times have you visited emergency rooms, excluding the Douglas, in the past 12 months?**

**□** 0=0

**□** 1=1

**□** 2=2

**□** 3=3

**□** 4=4

**□** 5=5

**□** 6=Other (Skip to 5.1)

***5.1:* Specify the number of times, if other:_________________**

**6. During the past 12 months, and before using emergency services at the Douglas, and at L’Autre Maison for that episode, have you used other services for your mental health problems, for alcohol or drug problems, or problems with your emotions?**

🞅 1= Yes

🞅 0= No (Skip to 6.2)

🞅 99= No answer (Skip to 6.2)

**I am going to present different types of services and ask you to indicate whether you have used any of them for your mental health problems, problems with alcohol or drugs, or problems with your emotions prior to using the Douglas Emergency and l’Autre Maison.**

***6.1 Have you been hospitalized (sent to a ward) for mental health, alcohol or drug problems?***

🞅1= Yes

🞅 0= No (Skip to 6.2)

🞅99= No answer (Skip to 6.2)

***6.1.1 How many times?***

***____________(specify number)***

***6.1.2 How satisfied were you with this service?***

🞅1= Not at all satisfied or dissatisfied

🞅2= Moderately satisfied

🞅3= Satisfied or completely satisfied

🞅99= No answer

***6.2 Do you have a family doctor?***

🞅1= Yes

🞅 0= No (Skip to 6.2.2)

🞅99= No answer

***6.2.1 Have you seen your family doctor?***

🞅1= Yes

🞅 0= No

🞅99= No answer

***6.2.1.1 How many times have you seen your family doctor?***

***_____________(specify number)***

***6.2.1.2 How satisfied were you with your family doctor?***

🞅1= Not at all satisfied or dissatisfied

🞅2= Moderately satisfied

🞅3= Satisfied or completely satisfied

🞅99= No answer

***6.2.2 Are you registered on a wait list for access to a family doctor?***

🞅1= Yes

🞅 0= No

🞅99= No answer

**6.3 *Have you had a consultation in a walk-in medical clinic with a doctor other than your family doctor?***

🞅1= Yes

🞅 0= No (Skip to 6.4)

🞅99= No answer

***6.3.1 How many times have you had a consultation in a walk-in medical clinic with a doctor other than your family doctor?***

***_______*** *(*specify number*)*

***6.3.2 How satisfied were you with this service?***

🞅1= Not at all satisfied, or dissatisfied

🞅2= Moderately satisfied

🞅3= Satisfied or completely satisfied

🞅99= No answer

***6.4 Have you consulted one or more psychiatrists outside of the emergency or a hospitalization?***

🞅1= Yes

🞅 0= No (Skip to 6.5)

🞅99= No answer

***6.4.1 How many times have you consulted one or more psychiatrists other than at the emergency or during a hospitalization?***

____________(specify number)

***6.4.2 How satisfied were you with this/these professional(s)?***

🞅1= Not at all satisfied, or dissatisfied

🞅2= Moderately satisfied

🞅3= Satisfied or completely satisfied

🞅99= No answer

**6.5 *Have you consulted the psychosocial team or specialized mental health team at a CLSC?***

🞅1= Yes

🞅 0= No (Skip to 6.6)

🞅99= No answer

***6.5.1 How many times?***

_______(specify number)

***6.5.2 How satisfied were you with this service?***

🞅1= Not at all satisfied, or dissatisfied

🞅2= Moderately satisfied

🞅3= Satisfied or completely satisfied

🞅99= No answer

**6.6 *Have you seen one or more psychologists in a private practice?***

🞅1= Yes

🞅 0= No (Skip to 6.7)

🞅99= No answer

***6.6.1 How many times?***

_______(specify number)

***6.6.2 How satisfied were you with this/these psychologists?***

🞅1= Not at all satisfied, or dissatisfied

🞅2= Moderately satisfied

🞅3= Satisfied or completely satisfied

🞅99= No answer

**6.7 *Have you received mental health, drug or alcohol, or other services from a community organization (e.g. crisis center, support services, counseling services, suicide prevention, addiction services)?***

🞅1= Yes

🞅 0= No (Skip to 6.8)

🞅99= No answer

***6.7.1 How many times?***

_______(specify number)

***6.7.2 How satisfied were you with this service?***

🞅1= Not at all satisfied, or dissatisfied

🞅2= Moderately satisfied

🞅3= Satisfied or completely satisfied

🞅99= No answer

**6.8 *Have you received other services?***

🞅1= Yes

🞅 0= No (Skip to 7)

🞅99= No answer

***6.8.1 Which one(s)? (specify)***

***_____________________________________________***

***6.8.1.1 How many times?***

_______(specify number)

***6.8.1.2 How satisfied were you with this/these service(s)?***

🞅1= Not at all satisfied, or dissatisfied

🞅2= Moderately satisfied

🞅3= Satisfied or completely satisfied

🞅99= No answer

**6.9 *Have you received other services?***

🞅1= Yes

🞅 0= No (Skip to 7)

🞅99= No answer

***6.9.1 Which?***

***_____________________________________________***

***6.9.1.1 How many times?***

_______ (specify)

***6.9.1.2 How satisfied were you with this/these service(s)?***

🞅1= Not at all satisfied, or dissatisfied

🞅2= Moderately satisfied

🞅3= Satisfied or completely satisfied

🞅99= No answer

**6.10 Have you received other services*?***

🞅1= Yes

🞅 0= No (Skip to 7)

🞅99= No answer

***6.10.1 Which?***

***_____________________________________________***

***6.10.1.1 How many times?***

_______(specify)

***6.10.1.2 How satisfied were you with this/these service(s)?***

🞅1= Not at all satisfied, or dissatisfied

🞅2= Moderately satisfied

🞅3= Satisfied or completely satisfied

🞅99= No answer

**6.11 Have you received other services?**

🞅1= Yes

🞅 0= No (Skip to 7)

🞅99= no answer

***6.11.1 Which?***

***_____________________________________________***

***6.11.1.1 How many times?***

_______(specify)

***6.11.1.2 How satisfied were you with this/these service(s)?***

🞅1= Not at all satisfied, or dissatisfied

🞅2= Moderately satisfied

🞅3= Satisfied or completely satisfied

🞅99= No answer

**7. I am going to read a sentence and ask you if you are: 1) not at all or slightly in agreement; 2) moderately in agreement; 3) in agreement or entirely in agreement, with this statement:**

***« Services outside of the emergency and L’Autre Maison meet my mental health needs. »***

🞅1= Not at all or slightly in agreement

🞅2= Moderately in agreement

🞅3= In agreement or entirely in agreement (Skip to 8)

🞅99= No answer (Skip to 8)

**7.1 Which of the following reasons explain why services outside of the emergency and L’Autre Maison do not meet or slightly meet, moderately meet, or entirely meet your mental health needs. Respond with a “yes” or “no” to the statements that I’m going to read:**

🞅1= You prefer to manage on your own

🞅2= You don`t know how, or where, to obtain the type of help appropriate to your problem

🞅3= You don`t find time to look after it (e.g. too busy)

🞅4= Your employment or occupation prevent you (e.g. workload, work schedule or lack of cooperation from the supervisor)

🞅5= The help isn`t readily available

🞅6= You don’t have confidence in the services.

🞅7= You don`t have the financial means.

🞅8= Your insurance doesn`t cover the costs.

🞅9= You are afraid of what others will think of you.

🞅10= There is a language problem.

🞅11= You prefer to count on your family or friends to help you.

🞅12 You are dissatisfied with the quality of services.

🞅13= Other (Skip to 7.1.1)

🞅99= No answer

***7.1.1 Specify any other reasons for which services outside of the emergency and L’Autre Maison do not meet, moderately meet, or entirely meet your mental health needs?***

***­­­­­­­­­­­­­­­­­­­__________________________________________________***

****Interviewer: Record the response here and on the server of your tablet.***

**8. How would you rate your general knowledge of mental health or addiction services?**

🞅1= Poor or very little knowledge

🞅 2= Moderate or good

🞅 3= Very good or excellent

🞅99= No answer

**B. QUALITY OF SERVICES RECEIVED AT THE EMERGENCY**

**9. Thinking of the Douglas Emergency, please indicate for each of the following statements, whether you are: 1= not at all or slightly in agreement; 2= moderately in agreement; 3= in agreement or entirely in agreement:**

|  | **1** | **2** | **3** | **97**  **Don’t know** | **99**  **No answer** |
| --- | --- | --- | --- | --- | --- |
| Service providers at the Douglas Emergency are respectful. |  |  |  |  |  |
| Service providers at the Douglas Emergency listen to my problems. |  |  |  |  |  |
| The information received at the Douglas emergency regarding my problems and treatments is sufficient. |  |  |  |  |  |
| I was adequately informed at the Douglas Emergency about the services available in my community that may address my needs. |  |  |  |  |  |
| My visits to the Douglas Emergency did not allow me to adequately discuss my problems. |  |  |  |  |  |

**10. Thinking of services received at the Douglas Emergency, would you say that you are:**

🞅1= Not at all or slightly satisfied

🞅2= Moderately satisfied

🞅3= Satisfied or completely satisfied

🞅99= No answer

**11. Thinking back to the way in which you were referred to the L’Autre Maison crisis center, would you say for each of the following statements that you are: 1) not at all or slightly in agreement; 2) moderately in agreement; or 3) in agreement or completely in agreement?**

|  | **1** | **2** | **3** | **99**  **No answer** |
| --- | --- | --- | --- | --- |
| A service provider at the Douglas Emergency explained to me the reasons for which I was referred to L’Autre Maison. |  |  |  |  |
| The information I received about L’Autre Maison was clear. |  |  |  |  |
| At the Douglas Emergency I was able to ask questions about the crisis services offered at L’Autre Maison. |  |  |  |  |
| I find that my referral to l’Autre Maison was fair. |  |  |  |  |

**C. QUALITY OF SERVICES RECEIVED AT THE L’AUTRE MAISON CRISIS CENTER**

**12. I am going to list the services offered by the L’Autre Maison crisis center and ask you to tell me if you have benefited from any of them after being referred there by the Douglas following this episode.**

***12.1. Did you receive lodging?***

1=Yes (Skip to 12.4)

0=No

99=No answer

***12.2. Have you received an intervention by telephone?***

1=Yes (skip to questions 12.5; 12.5.2)

0=No

99=No answer

***12.3 Have you received follow-up crisis services (including follow-up at the organization, or at home)?***

1=Yes (Skip to questions 2.6; 12.6.1; 12.6.3; 12.6.4)

0=No

99=No answer

***12.4 How long did you stay or receive lodging at l’Autre Maison following your episode at the emergency?***

_________________days; _________________ hours

***12.5 How many telephone interventions did you receive from l’Autre Maison following your episode at the emergency?***

**□** 0=0

**□** 1=1

**□** 2=2

**□** 3=3

**□** 4=4

**□** 5=Other (Skip to 12.5.1)

***12.5.1 Specify how many telephone interventions you have received from L’Autre Maison following your episode at the Emergency.***

***____________*** (number)

**12.5.2 How many minutes or hours did your telephone interventions with L’Autre Maison last on average?**

______________minutes: ______________ hours

**12.6 Did your follow-up crisis services take place at the organization or at home?**

**□**1= At the organization

**□**2= At my home

**12.6.1 How many follow-up crisis interventions have you received from L’Autre Maison following your visit to the Emergency?**

**□1=**1

**□2=**2

**□3=**3

**□4=**4

**□5=**5

**□6**=Other (Skip to 12.6.2)

***12.6.2 Specify how many follow-up crisis interventions you received from L’Autre Maison following your visit to the Emergency.***

**_________________** (number)

**12.6.3 How long did your crisis interventions last on average?**

______________minutes ______________ hours

**12.6.4 What was the total time period in which your crisis interventions occurred?**

Number of months:__________ weeks:____________

**13. Was this your first stay or contact with L’Autre Maison?**

 1= Yes

 0= No (Skip to 13.1)

 99= No answer

**13.1 How many times did you use the services of L’Autre Maison before your referral to this service by the emergency?**

**□** 0=0

**□** 1=1

**□** 2=2

**□** 3=3

**□** 4=4

**□** 5=Other (Skip to 13.1.1)

***13.1.1 How many times did you use the services of L’Autre Maison before your referral to this service by the Emergency?***

_____________ (number)

**14. Thinking of L’Autre Maison, would you say that you are: 1) not at all or slightly in agreement; 2) moderately in agreement; or 3) in agreement or complete agreement with each of the following statements:**

|  | **1** | **2** | **3** | **99**  **No answer** |
| --- | --- | --- | --- | --- |
| Service providers at L’Autre Maison are respectful. |  |  |  |  |
| Service providers at L’Autre Maison listen to my problems. |  |  |  |  |
| The information received at L’Autre Maison regarding my problems and treatments is sufficient. |  |  |  |  |
| My contact with L’Autre Maison allowed me to adequately resolve my problems. |  |  |  |  |
| At L’Autre Maison I was adequately informed about the services available in my community that may address my needs. |  |  |  |  |
| L’Autre Maison agreed to put me in contact with services or resources that will help my mental health. |  |  |  |  |
| L’Autre Maison allowed me to avoid returning to the Emergency for my mental health problems. |  |  |  |  |
| I can contact L’Autre Maison again anytime in case of problems. |  |  |  |  |
| I would not hesitate to recommend L’Autre Maison to any of my friends or close relations in a crisis. |  |  |  |  |

**15. Thinking of services you have received from L’Autre Maison, could you tell us whether you are:**

🞅1= Not at all or slightly satisfied

🞅2= Moderately satisfied

🞅3= Satisfied or completely satisfied

🞅99= No answer

**16. Have you stayed in contact with L’Autre Maison: that is, since your referral from the emergency to L’Autre Maison do you still use their crisis services on occasion?**

□1 Yes

□ 0 No (Skip to 17)

🞅98= Not applicable (Skip to 17)

🞅99= No answer (Skip to 17)

**16.1. Respond with a “yes”, or “no”, to the following statement: “Staying in contact with L’Autre Maison helps manage my mental health problems, emotional problems or problems with alcohol or drugs.”**

🞅1= Doesn’t help me at all, or helps very little

🞅2= Helps me somewhat

🞅3= Helps me a lot or completely

🞅99= No answer

**17. Since your meetings with L’Autre Maison, have you used services other than those from L’Autre Maison or the Douglas emergency for your mental health needs?**

□1 Yes

□ 0 No (Skip to 19)

🞅98= Not applicable (Skip to 19)

🞅99= No answer (Skip to 19)

**I am going to present different types of services, and ask you to indicate whether you have used them « after using L’Autre Maison ».**

***17.1 Your family doctor***

□ 1= Yes, and the referral or contact was arranged by L’Autre Maison

□ 2= Yes, but the referral or contact was not arranged by L’Autre Maison

□ 0= No, I have not used this service

🞅98= Not applicable

🞅99= No answer

***17.2 Another general practitioner at a walk-in medical clinic***

□ 1= Yes, and the referral or contact was arranged by L’Autre Maison

□ 2= Yes, but the referral or contact was not arranged by L’Autre Maison

□ 0= No, I have not used this service

🞅99= No answer

***17.3 Psychosocial or mental health services at a CLSC***

□ 1= Yes, and the referral or contact was arranged by L’Autre Maison

□ 2= Yes, but the referral or contact was not arranged by L’Autre Maison

□ 0= No, I have not used this service

🞅99= No answer

***17.4 Psychiatric services in a hospital (follow-up with a doctor or psychiatrist)***

□ 1= Yes, and the referral or contact was arranged by L’Autre Maison

□ 2= Yes, but the reference or contact was not arranged by L’Autre Maison

□ 0= No, I have not used this service

🞅99= No answer

***17.5 |Services for dealing with your problems related to consumption or gambling in an addiction rehabilitation center (CRD) or other community organization for addiction or gambling***

□ 1= Yes, and the referral or contact was arranged by L’Autre Maison

□ 2= Yes, but the referral or contact was not arranged by L’Autre Maison

□ 0= No, I have not used this service

🞅99= No answer

***17.6 Psychologist in private practice***

□ 1= Yes, and the referral or contact was arranged by L’Autre Maison

□ 2= Yes, but the referral or contact was not arranged by L’Autre Maison

□ 0= No, I have not used this service

🞅99= No answer

***17.7 Services at a crisis center***

□ 1= Yes, and the referral or contact was arranged by L’Autre Maison

□ 2= Yes, but the referral or contact was not arranged by L’Autre Maison

□ 0= No, I have not used this service

🞅99= No answer

***17.8 Mental health support services***

□ 1= Yes, and the referral or contact was arranged by L’Autre Maison

□ 2= Yes, but the referral or contact was not arranged by L’Autre Maison

□ 0= No, I have not used this service

🞅99= No answer

***17.9 Other community-based services***

□ 1= Yes, and the referral or contact was arranged by L’Autre Maison

□ 2= Yes, but the referral or contact was not arranged by L’Autre Maison

□ 0= No, I have not used this service

🞅99= No answer

***17.10 Other services***

□ 1= Yes, and the reference or contact was arranged by L’Autre Maison

□ 2= Yes, but the referral or contact was not arranged by L’Autre Maison

□ 0= No, I have not used this service Skip to 18)

🞅99= No answer (Skip to 18)

*17.10.1 What was this other service?*

*_____________________________________________________*

***17.11 Other services***

□ 1= Yes, and the referral or contact was arranged by L’Autre Maison

□ 2= Yes, but the referral or contact was not arranged by L’Autre Maison

□ 0= No, I have not used this service Skip to 18)

🞅99= No answer (Skip to 18)

*17.11.1 What was this other service?*

________________________________________________________

**18. I am going to read a statement and ask you to tell me whether you are: 1) not at all or slightly in agreement, 2) moderately in agreement; or 3) in agreement or entirely in agreement: “The services I used after my stay at L’Autre Maison responded to my needs.”**

🞅1= Not at all or slightly in agreement

🞅2= Moderately in agreement

🞅3= In agreement or entirely in agreement

🞅99= No answer

**19. If you have not used services for your mental health problems, emotions, or problems related to alcohol or drugs since obtaining support from L’Autre Maison, would you have need of such services to help you?**

□ 1= Yes

□ 0= No

🞅99= No answer

**D. SOCIO-DEMOGRAPHIS AND CLINICAL DATA**

**20. How old are you? ________ years**

**21. Are you?**

**□** 1= a man

**□** 0= a woman

**□** 2 = Other (Skip to 21.1)

*21.1 How would you describe you gender?*

________________________________

**22. I am going to list some types of lodging; tell me in which one you are living.**

**□** 1= Residence such as a house, condo, or rental apartment

**□** 2= Supervised apartment

**□** 3= Subsidized apartment (e.g. H.L.M. or O.S.B.L)

**□** 4= Family-type residence

**□** 5= Group home

**□** 6= No fixed residence

**□** 7= Other (Skip to 22.1)

**□** 99 = No answer

*22.1 In what type of lodging are you living, if other?*

______________________________________

**23. What is your marital status?**

**□** 1= Single

**□** 2= Separated/ divorced/ widowed

**□** 3= Married/ common law

**□** 4= Other (Skip to 23.1)

**□** 99= No answer

*23.1 What is your marital status, if other?*

_______________________________________

**24. Do you have children?**

**□** 1= Yes, and have custody

**□** 2= Yes, but not custody

**□** 0= No

**□** 99= No answer

**25. Do you have close friends on whom you can rely in case of need?**

**□** 1= Yes

□ 0= No (Skip to 25.1)

□ 99= No answer

***25.1 How many close friends do you have, on whom you can rely in case of need?***

**_____________________________________________**

**26. What is your highest level of education?**

**□** 1= Elementary school

**□** 2= High school

**□**3= CEGEP or more

**□** 4= No schooling

**□** 99= No answer

**27. Are you currently working?**

**□** 1= Yes, full time

**□** 2= Yes, part time

**□** 0= No (answer question 27.1)

**□** 98= Not applicable

**□** 99 = No answer

**27.1 If not working, what is your current situation?**

**□**1= Student

**□**2= On unemployment insurance, or the CNESST (illness insurance)

**□** 3= On unemployment insurance or social assistance

**□** 4= Retired or annuity

**□** 5= without personal income

**□** 6= Other (respond to question 27.1.1)

**□** 99 = No answer

**CNESST: Commission des normes, de l’équité, de la santé et de la sécurité du travail*

**27.1.1 What is your current situation?**

*_________________________________________*

**28. In your opinion, your physical health is:**

🞅 1= Poor or fair

🞅 2= Moderately good or good

🞅 3= Very good or excellent

🞅 99= No answer

**29. In your opinion, your mental health is:**

🞅 1= Poor or fair

🞅 2= Moderately good or good

🞅 3= Very good or excellent

🞅 99= No answer

**30. Have you received one or more mental health diagnoses in the past 12 months, or a previous diagnosis that persists?**

□ 1= Yes (respond to question 30.1)

□ 0= No

□ 99= No answer

**30.1 If yes, please identify which mental health diagnosis/es you have received in the past 12 months, or previous diagnoses that persist:**

**~~---------------------------------------~~**

**31. Have you had problems with alcohol consumption in the past 12 months?**

□ 1= Yes

□ 0= No

□ 99= No answer

**32. Have you had problems with drug in the past 12 months?**

□ 1= Yes (respond to question 32.1)

□ 0= No

□ 99= No answer

*32.1 If yes, please indicate which type of drug(s):* ***_________________________***

**33. Have you had gambling problems in the past 12 months?**

□ 1= Yes

□ 0= No

□ 99= No answer

**34. Have you experienced suicidal ideation in the past 12 months?**

□ 1= Yes

□ 0= No

□ 99= No answer

**35. What was your family income in the past year? _________________$**

***35.1 I am going to present some annual salary categories and ask you to tell me which one corresponds best to your family income:***

**□** 1= 0 à 9 999$/year

**□** 2= 10 000 à 19 999$/year

**□** 3= 20 000 à 29 999$/year

**□** 4= 30 000 à 39 999$/year

**□** 5= 40 000 à 49 999$/year

**□** 6= 50 000 à 59 999$/year

**□** 7= 60 000 à 69 999$/year

**□** 8= 70 000 à 79 999$/year

**□** 9= 80 000 à 89 999$/year

**□** 10= 90 000 à 99 999$/year

**□** 11= 100 000$ et plus/year

**□** 99= No answer

**E – QUALITATIVE QUESTIONS**

**36. Recalling the overall service provided by L’Autre Maison, tell me in a few words what you most appreciated.**

*****Interviewer: record the response of the patient below, and also provide a written summary on your tablet.***

**___________________________________________________________________________**

**37. Tell me what could be improved in terms of the services offered by L’Autre Maison?**

*****Interviewer: record the response of the patient below, and also provide a written summary on your tablet.***

**___________________________________________________________________________**

**38. Other than L’Autre Maison, what would most help you to improve your overall mental health problems and better pass through periods of crisis?**

****** *Interviewer: record the response of the patient below, and also provide a written summary on your tablet.***

**We thank you kindly for your participation in our project. Your collaboration is invaluable and will contribute to our recommendations aimed at better responding to your needs!**

**Questionnaire for AMI-Quebec peer supporters on the quality of support offered and service coordination**

Interview date:

User code (peer supporters):

Interviewer’s name:

**Introduction**

You went to the Douglas emergency department with your close relation at some time during the **last 3 months**, and were referred to an **AMI-Quebec** peer supporter. I will ask you about your visit to the emergency department (all services) and services received by your close relation, as well as your situation and that of your close relation and the services you are receiving from AMI-Quebec. These questions will help us understand how you and that your close relation used the services and your satisfaction with the services received, in order to make recommendations for improving mental health services.

**A. USE OF EMERGENCY SERVICES AND GENERALLY**

**1. In a few words, why did you and your close relation go to the Douglas emergency department, at the time of your referral to the services of AMI-Quebec? (Here the interviewer specifies the referral date given by AMI-Quebec.)**

___________________________________

****The interviewer records the patient’s response, and also summarizes it in writing on the server of his tablet.***

**2. How long did you stay in the emergency department with your close relation on…? (The interviewer states the date of the visit.)**

**_______minutes or _______hours**

- 1. **And how long did you stay in the emergency department with your close relation before meeting with the AMI-Quebec worker?**

**_______minutes or _______hours or ________ days**

**3. During the past 12 months and including the visit on _________ (to be specified by the interviewer), how often did you accompany your close relation to the Douglas emergency department for mental health or substance use reasons?**

**□** 1 = 1

**□** 2 = 2

**□** 3 = 3

**□** 4 = 4

**□** 5 = 5

**□** 6 = Other (Go to item 3.1)

***3.1 Specify the number of times***

_________________

**4. Outside of the Douglas emergency department, has your close relation visited other emergency departments in the past 12 months?**

🞅 1 = Yes

🞅0 = No (Go to item 5)

97 = Don’t know (Go to item 5)

99 = No answer (Go to item 5)

***4.1 How many times have they visited emergency departments other than Douglas in the past 12 months?***

**□** 1 = 1

**□** 2 = 2

**□** 3 = 3

**□** 4 = 4

**□** 5 = 5

□ 6 = Other (Go to item 4.1.1)

*4.1.1 Specify the number of times*

_________________

*4.1.2 Globally, for how many visits have you accompanied your close relation in the last 12 months?*

**□** 0 = 0

**□** 1 = 1

**□** 2 = 2

**□** 3 = 3

**□** 4 = 4

**□** 5 = 5

□ 6 = Other (Go to item 4.1.2.1)

*4.1.2.1 Specify the number of times*

_________________

**5. During the past 12 months, and before using the Douglas emergency services that led to your support by AMI-Quebec, did your close relation use other services for their mental health problems, alcohol or drug use, or emotional issues?**

🞅 1 = Yes

🞅 0 = No *(Go to item 7)*

🞅 97 = Don’t know *(Go to item 7)*

🞅 99 = No answer *(Go to item 7)*

**6.** **I am going to mention various types of services that your close relation may have used before the visit to the Douglas emergency department that led to your follow-up with AMI-Quebec, for their mental health problems, alcohol or drug use, or emotional issues. In the last 12 months:**

**6.1 *Were they hospitalized (in a ward) for their mental health problems, alcohol or drug use, or emotional issues?***

🞅1 = Yes

🞅0 = No *(Go to item 6.2)*

🞅97 = Don’t know *(Go to item 6.2)*

🞅99 = No answer *(Go to item 6.2)*

***6.1.1 How many times?***

***____________number of times***

***6.1.2 How satisfied were you with this service?***

🞅1 = Not at all or not very satisfied

🞅2 = Moderately satisfied

🞅3 = Satisfied or completely satisfied

🞅99 = No answer

**6.2 Do they have a family doctor?**

🞅1 = Yes

🞅0 = No *(Go to item 6.2.2)*

🞅97 = Don’t know *(Go to item 6.3)*

**6.2.1 Did they see their family doctor?**

🞅1 = Yes

🞅0 = No *(Go to item 6.2.2)*

🞅97 = Don’t know *(Go to item 6.3)*

**6.2.1.1 How many times did they see their family doctor?**

***____________number of times***

**6.2.1.2 How satisfied were you with your family doctor?**

🞅1 = Not at all or not very satisfied

🞅2 = Moderately satisfied

🞅3 = Satisfied or completely satisfied

🞅99 = No answer

**6.2.2 Are they on a waiting list to access a family doctor?**

🞅1 = Yes

🞅0 = No

🞅97 = Don’t know

**6.3 *Did they go to a walk-in medical clinic to see a doctor other than their family doctor if necessary?***

🞅1 = Yes

🞅0 = No *(Go to item 6.4)*

🞅97 = Don’t know *(Go to item 6.4)*

***6.3.1 How many times did they go to a walk-in medical clinic to see a doctor other than their family doctor?***

***_______number of times***

***6.3.2 How satisfied were you with this service?***

🞅1 = Not at all or not very satisfied

🞅2 = Moderately satisfied

🞅3 = Satisfied or completely satisfied

🞅99 = No answer

***6.4 Did they see a psychiatrist or psychiatrists outside the emergency department or as an outpatient?***

🞅1 = Yes

🞅0 = No *(Go to item 6.5)*

🞅97 = Don’t know *(Go to item 6.5)*

***6.4.1 How many times did they see a psychiatrist or psychiatrists other than in an emergency department or as an outpatient?***

____________number of times

***6.4.2 What was your level of satisfaction with these professional services?***

🞅1 = Not at all or not very satisfied

🞅2 = Moderately satisfied

🞅3 = Satisfied or completely satisfied

🞅99 = No answer

**6.5 *Did they meet with the psychosocial or specialized mental health team at the CLSC?***

🞅1 = Yes

🞅0 = No *(Go to item 6.6)*

🞅97 = Don’t know *(Go to item 6.6)*

***6.5.1 How many times?***

_______number of times

***6.5.2 How satisfied were you with this service?***

🞅1 = Not at all or not very satisfied

🞅2 = Moderately satisfied

🞅3 = Satisfied or completely satisfied

🞅99 = No answer

**6.6 *Did they see one or more psychologists in a private practice?***

🞅1 = Yes

🞅0 = No *(Go to item 6.7)*

🞅97 = Don’t know *(Go to item 6.7)*

***6.6.1 How many times?***

_______number of times

***6.6.2 How satisfied were you with this service?***

🞅1 = Not at all or not very satisfied

🞅2 = Moderately satisfied

🞅3 = Satisfied or completely satisfied

🞅99 = No answer

**6.7 *Have they received services from a community organization (e.g. crisis centre, self-help, helpline, suicide prevention, addiction)?***

🞅1 = Yes

🞅0 = No *(Go to item 6.8)*

🞅97 = Don’t know *(Go to item 6.8)*

***6.7.1 How many times?***

_______number of times

***6.7.2 How satisfied were you with such services?***

🞅1 = Not at all or not very satisfied

🞅2 = Moderately satisfied

🞅3 = Satisfied or completely satisfied

🞅99 = No answer

**6.8 *Did they receive other services?***

🞅1 = Yes

🞅0 = No *(Go to item 7)*

🞅97 = Don’t know *(Go to item 7)*

***6.8.1 Which ones?***

***_____________________________________________***

***6.8.1.1 How many times?***

_______number of times

***6.8.1.2*** ***How satisfied were you these services?***

🞅1 = Not at all or not very satisfied

🞅2 = Moderately satisfied

🞅3 = Satisfied or completely satisfied

🞅99 = No answer

**6.9 *Did they receive other services?***

🞅1 = Yes

🞅0 = No *(Go to item 7)*

🞅97 = Don’t know *(Go to item 7)*

***6.9.1 Which ones?***

***_____________________________________________***

***6.9.1.1 How many times?***

_______number of times

***6.9.1.2*** ***How satisfied were you these services?***

🞅1 = Not at all or not very satisfied

🞅2 = Moderately satisfied

🞅3 = Satisfied or completely satisfied

🞅99 = No answer

**6.10 *Did they receive other services?***

🞅1 = Yes

🞅0 = No *(Go to item 7)*

🞅97 = Don’t know *(Go to item 7)*

***6.10.1 Which ones?***

***_____________________________________________***

***6.10.1.1 How many times?***

_______number of times

***6.10.1.2*** ***How satisfied were you these services?***

🞅1 = Not at all or not very satisfied

🞅2 = Moderately satisfied

🞅3 = Satisfied or completely satisfied

🞅99 = No answer

**6.11 *Did they receive other services?***

🞅1 = Yes

🞅0 = No *(Go to item 7)*

🞅97 = Don’t know *(Go to item 7)*

***6.11.1 Which ones?***

***_____________________________________________***

***6.11.1.1 How many times?***

_______number of times

***6.11.1.2 How satisfied were you these services?***

🞅1 = Not at all or not very satisfied

🞅2 = Moderately satisfied

🞅3 = Satisfied or completely satisfied

🞅99 = No answer

***7.* I will read you a sentence and please tell me if you 1 = Totally or mostly disagree; 2 = Moderately agree; 3 = Agree or totally agree: *“Services received outside the emergency department meet the mental health needs of your close relation.”***

🞅 1 = Totally or mostly disagree

🞅 2 = Moderately agree

🞅 3 = Agree or totally agree *(Go to item 8)*

98 = N/A *(Go to item 8)*

🞅99 = No answer *(Go to item 8)*

**7.1 What are the reasons why non-emergency services do not or only slightly or moderately meet your close relation’s needs? Answer yes or no to the statements I am going to read to you:**

🞅 1 = They prefer to fend for themselves

🞅 2 = We do not know how or where to get the right kind of help for their problems

🞅 3 = They don’t have time to take care of it (e.g. too busy)

🞅 4 = Their job or occupation prevents them from getting help (e.g. workload, work hours or lack of cooperation from the supervisor)

🞅 5 = Help is not readily available

🞅 6 = They do not trust the services

🞅 7 = They lack the financial means

🞅 8 = Their insurance (or yours) does not cover their care expenses

🞅 9 = They fear what others may think

🞅 10 = There is a language problem

🞅 11 = They prefer to rely on you, their family or friends for help

🞅 12 = They are dissatisfied with the quality of services

🞅 13 = Other *(Go to item 7.1.1)*

🞅 99 = No answer

***7.1.1 What are the other reasons why non-emergency services do not or only slightly or moderately meet your close relation’s needs?***

***______________________________________________***

**The interviewer records the question and answer on the tablet server*

**8. Apart from the services offered by AMI-Quebec, have you used other support services in the last 12 months?**

🞅 1 = Yes

🞅 0 = No *(Go to item 8.4)*

🞅 99 = No answer *(Go to item 8.4)*

**8.1 Can you tell me what service you use?**

***Service #1: __________________________***

***8.1.1 Was the service used linked to a referral or contact established by AMI-Quebec?***

****The interviewer takes each of the services listed by the participant and asks them whether or not the service used was linked to a referral or contact established by AMI-Quebec.***

□ 1 = Yes, referral or contact established by AMI-Quebec

□ 0 = Not established or referred by AMI-Quebec

***8.2* Can you tell me what service you use?**

***Service #2: __________________________***

** Interviewer enters 98 if no service*

***8.2.1*** ***Was the service used linked to a referral or contact established by AMI-Quebec***

□1 = Yes, referral or contact established by AMI-Quebec

□ 0 = Not established or referred by AMI-Quebec

***8.3* Can you tell me what service you use?**

***Service #3: __________________________***

***8.3.1*** ***Was the service used linked to a referral or contact established by AMI-Quebec?***

□ 1 = Yes, referral or contact established by AMI-Quebec

□ 0 = Not established or referred by AMI-Quebec

**8.4 In your opinion, your knowledge of mental health or addiction services is:**

🞅 1 = Poor or fair

🞅 2 = Average or good

🞅 3 = Very good or excellent

🞅99 = No answer

**8.5** **In your opinion, your close relation’s knowledge of mental health or addiction services is:**

🞅 1 = Poor or fair

🞅 2 = Average or good

🞅 3 = Very good or excellent

🞅 99 = No answer

**B. QUALITY OF SERVICE RECEIVED AT EMERGENCY GLOBALLY OR BY AMI-QUEBEC IN EMERGENCY**

**9. Approximately how long did the meeting with AMI-Quebec’s emergency worker last?**

🞅__________ minutes or

🞅 __________ hours

**10. After first meeting with AMI-Quebec’s emergency worker, did you contact them again? Answer one of the following choices:**

🞅 1 = Yes, and I am satisfied with their intervention

🞅 2 = Yes, and I am not satisfied with their intervention

🞅 3 = No, I did not need it

🞅4 = No, I was not up to it

🞅 99 = No answer

**11. Thinking about emergency personnel generally, as well as the AMI-Quebec emergency worker, for each of the following statements, could you tell us if you 1 = Totally or mostly disagree; 2 = Moderately agree; 3 = Agree or totally agree:**

|  | **1** | **2** | **3** | **99**  **No answer** | **98**  **N/A** |
| --- | --- | --- | --- | --- | --- |
| a. Emergency workers at the Douglas are respectful. |  |  |  |  |  |
| a1. Similarly, for the AMI-Quebec worker. |  |  |  |  |  |
| b. Douglas emergency workers are responsive to our problems. |  |  |  |  |  |
| a1. Similarly, for the AMI-Quebec worker. |  |  |  |  |  |
| c. The information received at the Douglas emergency about problems and treatments is sufficient. |  |  |  |  |  |
| c1. The same applies to the information received from the AMI-Quebec worker. |  |  |  |  |  |
| d. At the Douglas emergency, I was informed adequately about the services available in the community to meet my needs or those of my close relation. |  |  |  |  |  |
| c1. The same applies to the information received from the AMI-Quebec worker. |  |  |  |  |  |
| e. Visits to the Douglas emergency allowed my close relation to adequately deal with their problems. |  |  |  |  |  |
| e1. Similarly, the AMI-Quebec emergency worker, responded adequately to my requests and problems. |  |  |  |  |  |

**12. Thinking about the services that your close relation received at the Douglas emergency, could you tell us if you are:**

🞅 1 = Not satisfied at all or not very satisfied

🞅 2 = Moderately satisfied

🞅 3 = Satisfied or completely satisfied

🞅 99 = No answer

**13. Thinking about the emergency services provided to you by AMI-Quebec, could you tell us if you are:**

🞅 1 = Not satisfied at all or not very satisfied

🞅 2 = Moderately satisfied

🞅 3 = Satisfied or completely satisfied

🞅 99 = No answer

**14. Thinking about how you were referred to AMI-Quebec’s services at the Douglas emergency, could you tell us, for each of the following statements, if you 1 = Totally or mostly disagree; 2 = Moderately agree; 3 = Agree or totally agree:**

|  | **1** | **2** | **3** | **99**  **No answer** |
| --- | --- | --- | --- | --- |
| a. An emergency worker from the Douglas explained to me why I was being referred to AMI-Quebec. |  |  |  |  |
| b. The information provided at the Douglas emergency about AMI-Quebec was clear. |  |  |  |  |
| c. At the Douglas emergency, I was able to ask questions about AMI-Quebec’s services. |  |  |  |  |
| d. I think it was appropriate to refer me to AMI-Quebec. |  |  |  |  |

**15. Did the AMI-Quebec emergency worker refer you to services for caregivers?**

🞅 1 = Yes

🞅 0 = No (Go to Section D)

***15.1 What is the name of this resource?***

**** May also be AMI-Quebec***

**16. Did you contact and use the services of the caregiver resource to which AMI-Quebec referred you at the emergency department?**

🞅 1 = Yes

🞅 0 = No (Go to Section D)

🞅 99 = No answer (Go to Section D)

**C. QUALITY OF SERVICES RECEIVED BY AMI-QUEBEC OR OTHER PEER-HELP RESOURCES FOR FAMILIES**

**17. What types of support did you receive from AMI-Quebec or from the resource indicated above (at the emergency department or afterwards with the AMI-Quebec resource)?**

****Several answers are possible.***

*****The interviewer asks the participant to answer yes or no, as the items are stated.***

🞅 1 = Individual psychosocial support to help me (e.g. I was heard; I was given some advice or ways of doing things to feel better, and to better manage my problems or those of my close relation)

🞅 2 = Group support for you and your close relation who has a mental health disorder (e.g. participation in support groups on anxiety, bipolarity, depression, etc.) offered by AMI-Quebec directly

🞅 3 = Group support for you only offered by AMI-Quebec directly

🞅 4 = Group support for my close relation offered by AMI-Quebec directly

🞅 5 = Respite and emergency care for my close relation (to free myself a little)

🞅 6 = I was referred to receive help from psychosocial or mental health services in my neighbourhood CLSC

🞅 7 = I was referred to the CLSC mental health services, for my close relation only

🞅 8 = I was referred to other community organizations that could help my close relation (e.g. crisis centre, self-help group)

🞅 9 = I was referred to an addiction rehabilitation centre for my close relation

🞅 10 = I was referred to another association for families whose relatives have a mental health problem

🞅 11 = I was referred to another resource whose mission is related to my needs

🞅 12 = Other for oneself (Go to item 17.1)

🞅 13 = Other for my close relation (Go to item 17.2)

🞅 99 = No answer

***17.1 What was this support (for you)?***

***17.2 What was this support (for your close relation)?***

**18. Thinking about AMI-Quebec for each of the following statements, could you tell us if you 1 = Totally or mostly disagree; 2 = Moderately agree; 3 = Agree or totally agree:**

|  | **1** | **2** | **3** | **97**  **Don’t know** | **98**  **N/A** | **99**  **No answer** |
| --- | --- | --- | --- | --- | --- | --- |
| a. I received sufficient information from AMI-Quebec about the services available in mental health for my close relation or to support me. |  |  |  |  |  |  |
| b. The support I received from AMI-Quebec allowed me to adequately deal with my problems related to my close relation. |  |  |  |  |  |  |
| c. AMI-Quebec has put me in touch with workers or resources that will help me and my close relation. |  |  |  |  |  |  |
| d. AMI-Quebec has helped my close relation in no longer having to call on an emergency department to deal with their mental health problems. |  |  |  |  |  |  |
| e. I can again contact AMI-Quebec at any time if a problem occurs. |  |  |  |  |  |  |
| f. I would not hesitate to recommend AMI-Quebec to any acquaintance whose relative has a mental health problem. |  |  |  |  |  |  |

**18.1 I will read you a sentence and please tell me if you 1 = Totally or mostly disagree; 2 = Moderately agree; 3 = Agree or totally agree. The services of AMI-Quebec or the resource indicated above have met my needs.**

🞅 1 = Totally or mostly disagree

🞅 2 = Moderately agree

🞅 3 = Agree or totally agree (Go to item 19)

🞅 99 = No answer (Go to question 19)

**18.2 Since this resource did not meet your needs, or only met them slightly or partially, can you explain the main reason, among the following choices of answers?**

🞅 1 = The mission of the association or organization was not related to my needs

🞅 2 = The approach of the association or organization does not suit me

🞅 3 = The structure of this association or organization does not suit me

🞅 4 = The services offered by this association or organization do not suit me

🞅 5 = Other (Go to item 18.2.1)

***18.2.1 What was the other main reason why the resource did not meet your needs or met them only slightly or partially?***

**The interviewer records the question and answer on the tablet server.*

**D. SOCIO-DEMOGRAPHIC AND CLINICAL DATA**

**19. How old are you?** **_________ years**

**20. You are?**

**□** 1 = A man

**□** 0 = A woman

**□** 2 = Other (Go to item 20.1)

*20.1 How do you define your gender?*

___________________________________

**21. I will give you some types of housing, tell me which one describes where you live.**

**□** 1 = House, condo, rental apartment

**□** 2 = Supervised apartment

**□** 3 = Subsidized housing (e.g. public or non-profit):

**□** 4 = Foster family

**□** 5 = Group home

**□** 6 = Homeless

□ 7 = Other (Go to item 21.1)

**□** 99 = No answer

*21.1 In what other type of dwelling do you live?*

______________________________________________________

**22. What is your marital status?**

**□** 1 = Single

**□** 2 = Separated/divorced/widowed

**□** 3 = Common law/married

**□** 4 = Other (Go to item 22.1)

**□** 99 = No answer

*22.1 What is your marital status if other?*

_______________________________________

**23. Do you have children?**

**□** 1 = Yes and dependent

**□** 2 = Yes, but not dependent

**□** 0 = No

**□** 99 = No answer

**24. Do you have close friends on whom you can rely when needed?**

**□** 1 = Yes (Go to item 24.1)

**□** 0 = No

**□** 99 = No answer

**24.1. How many close friends do you have?**

__________________

**25. What is your level of education?**

**□** 1 = Primary

**□** 2 = Secondary

**□** 3 = College or higher

**□** 4 = No schooling

**□** 99 = No answer

**26. Are you working right now?**

**□** 1 = Yes, full time

**□** 2 = Yes, part-time

□ 0 = No (Go to item 26.1)

**□** 98 = N/A

**□** 99 = No answer

**26.1 If no, what is your current situation?**

**□** 1 = Student

**□** 2 = On unemployment insurance, or CNESST (worker’s compensation)

**□** 3 = On income security (or social welfare)

**□** 4 = Retired or pensioner

**□** 5 = Without personal income

**□** 6 = Other (Go to item 26.1.1)

**□** 99 = No answer

**CNESST: Commission des normes, de l’équité, de la santé et de la sécurité du travail*

**26.1.1 What is your current situation if other?**

*_________________________________________*

**27. In your opinion, your physical health is:**

🞅 1 = Poor or fair

🞅 2 = Average or good

🞅 3 = Very good or excellent

🞅99 = No answer

**28. In your opinion, your mental health is:**

🞅 1 = Poor or fair

🞅 2 = Average or good

🞅 3 = Very good or excellent

🞅 99 = No answer

**29. Have you been diagnosed with a mental health problem or addiction disorder in the past 12 months or ongoing?**

□ 1 = Yes (Go item 29.1)

□ 0 = No

□ 99 = No answer

***29.1 If yes, can you tell us what diagnoses of mental disorders you have received in the past 12 months or that are ongoing?***

**30. Have you had problems with alcohol abuse in the last 12 months?**

□ 1 = Yes

□ 0 = No

**□** 99 = No answer

**31. Have you had problems related to drug use in the last 12 months?**

□ 1 = Yes (Go to item 31.1)

□ 0 = No

**□** 99 = No answer

***31.1 If yes, can you tell us what type of drug? _________________________***

**32. Have you had problems related to gambling in the last 12 months?**

□ 1 = Yes

□ 0 = No

**□** 99 = No answer

**33. Have you had any suicidal thoughts in the last 12 months?**

□ 1 = Yes

□ 0 = No

**□** 99 = No answer

**34. In the last year, what was your family income? _________________$**

***If the respondent cannot specify their family income, invite them to do so according to the following categories:***

***34.1 I will present annual salary categories; please tell me which one best describes your family income.***

**□** 1 = $0 to $9,999/year

**□** 2 = $10,000 to $19,999/year

**□** 3 = $20,000 to $29,999/year

**□** 4 = $30,000 to $39,999/year

**□** 5 = $40,000 to $49,999/year

**□** 6 = $50,000 to $59,999/year

**□** 7 = $60,000 to $69,999/year

**□** 8 = $70,000 to $79,999/year

**□** 9 = $80,000 to $89,999/year

**□** 10 = $90,000 to $99,999/year

**□** 11 = $100,000 and over/year

**□** 99 = No answer

**** The interviewer shows the card on income categories to participants.***

**E. SOCIO-DEMOGRAPHIC AND CLINICAL DATA *FOR THE CLOSE RELATION***

**35. What is your close relation’s age?**

**_________ years**

**36. Your close relation is?**

**□** 1 = A man

**□** 0 = A woman

□ 2 = Other (Go to item 36.1)

***36.1 How do they define their gender if other?***

*_______________________________*

**37. I will mention types of housing; please tell me where your close relation lives.**

**□** 1 = House, condo, rental apartment

**□** 2 = Supervised apartment

**□** 3 = Subsidized housing (e.g. public or non-profit):

**□** 4 = Foster family

**□** 5 = Group home

**□** 6 = Homeless

**□** 7 = Other (Go to item 37.1)

**□** 97 = Don’t know

***37.1 In what other type of dwelling do they live?***

______________________________________________________

**38. What is the marital status of you close relation?**

**□** 1 = Single

**□** 2 = Separated/divorced/widowed

**□** 3 = Common law/married

**□** 4 = Other (Go to item 38.1)

**□** 97 = Don’t know

***38.1 What is their marital status if other?***

_______________________________________

**39. Does your close relation have children?**

**□** 1 = Yes and dependent

**□** 2 = Yes, but not dependent

**□** 0 = No

**□** 97 = Don’t know

**40. Does your close relation have close friends that they can rely on?**

□ 1 = Yes (Go to item 40.1)

□ 0 = No

□ 97 = Don’t know

***40.1. How many close friends does your close relation have that they can rely on when needed?***

__________________

**41. What is your close relation’s level of education?**

**□** 1 = Primary

**□** 2 = Secondary

**□** 3 = College or higher

**□** 4 = No schooling

**□** 97 = Don’t know

**42. Is your close relation currently working?**

**□** 1 = Yes, full time

**□** 2 = Yes, part-time

**□** 0 = No (Go to item 42.1)

**□** 97 = Don’t know

**□** 98 = N/A

***42.1 If no, what is their current situation?***

**□** 1 = Student

**□** 2 = On unemployment insurance, or CNESST (worker’s compensation)

**□** 3 = On income security (or social welfare)

**□** 4 = Retired or pensioner

**□** 5 = Without personal income

**□** 6 = Other (Go to item 42.1.1)

**□** 97 = Don’t know

**CNESST: Commission* des normes, de l’équité, de la santé et de la sécurité du travail

***42.1.1 What is their current situation if other?***

*_________________________________________*

**43. Your close relation’s physical health is:**

🞅 1 = Poor or fair

🞅 2 = Average to good

🞅 3 = Very good or excellent

🞅97 = Don’t know

**44. Your close relation’s mental health is:**

🞅 1 = Poor or fair

🞅 2 = Average to good

🞅 3 = Very good or excellent

🞅97 = Don’t know

**45. Has your close relation been diagnosed with any mental disorders in the past 12 months or ongoing?**

□ 1 = Yes (Go to item 45.1)

□ 0 = No

**□** 97 **=** Don’t know

***45.1 If yes, can you tell us what diagnoses of mental disorders they have received in the past 12 months or that are ongoing?***

_

**46. Did your close relation have any drinking problems in the last 12 months?**

□ 1 = Yes

□ 0 = No

**□** 97 = Don’t know

**47. Did your close relation have any drug problems in the last 12 months?**

□ 1 = Yes (Go to item 47.1)

□ 0 = No

**□** 97 = Don’t know

***47.1 If yes, can you tell us what type of drug? _________________________***

**48. Did your close relation have any gambling problems in the last 12 months?**

□ 1 = Yes

□ 0 = No

**□** 97 = Don’t know

**49. Did your close relation have any suicidal ideation in the last 12 months?**

□ 1 = Yes

□ 0 = No

**□** 97 = Don’t know

**50. What was you close relation’s family income in the past year? _________________$**

****If the respondent cannot specify their family income, invite them to do so according to the following categories:***

***50.1*** ***I will present annual salary categories; please tell me which one best describes your close relation’s family income.***

**□** 1 = $0 to $9,999/year

**□** 2 = $10,000 to $19,999/year

**□** 3 = $20,000 to $29,999/year

**□** 4 = $30,000 to $39,999/year

**□** 5 = $40,000 to $49,999/year

**□** 6 = $50,000 to $59,999/year

**□** 7 = $60,000 to $69,999/year

**□** 8 = $70,000 to $79,999/year

**□** 9 = $80,000 to $89,999/year

**□** 10 = $90,000 to $99,999/year

**□** 11 = $100,000 and over/year

**□** 97 = Don’t know

**** The interviewer shows the card on income categories to participants.***

**F- QUALITATIVE QUESTIONS**

**51. In general, thinking back to AMI-Quebec’s services, tell me in a few words what you liked the most.**

**___________________________________________________________________________**

**52. Tell me now what aspects of AMI-Quebec’s services could be improved.**

**___________________________________________________________________________**

**53. Apart from the support received by AMI-Quebec, what helps you the most to cope with your situation?**

**Our warmest thanks for your participation in this project.**

**Your collaboration is valuable and will contribute to our recommendations**

**to better meet your**

**Interview guide (focus groups)**

**The interview guides are only available in French.**

**Guide d’entrevue: Relance**

**A. Présentation des personnes présentes**

1. Présentation et tour de table: nom & description du poste occupé

**B. Contexte d’implantation de la Relance**

1. Pouvez-vous nous présenter le ***contexte d’implantation*** de la Relance ?
   1. ***Année d’implantation*** ?
   2. Ce qui ***justifie la création*** de la Relance ?
   3. ***Évolution/transformation*** au cours des mois/années ?
   4. ***Difficultés d’implantation*** rencontrées et ***forces*** du processus d’implantation
   5. ***Formalisation du processus*** s’il y a lieu ? ***Procédures*** de formalisation mises en place ? Ce qui est ici à ***améliorer*** ?
2. Comment s’effectuent les liens entre l’***urgence et la Relance*** ?
   1. Comment est ***identifié un patient*** ***à l’urgence*** pour référence à la Relance ?
   2. Comment se fait le ***processus de référence*** du patient à la Relance ?
   3. Commentez les ***conditions de succès*** ou les ***difficultés*** des relations et modes de fonctionnement entre l’urgence et la Relance ?
   4. Au cours du processus d’implantation de la Relance, comment les relations avec les intervenants de l’urgence et de la Relance se sont-elles ***modifiées*** ?
   5. Quelles seraient les ***recommandations pour un succès optimal*** des relations et procédures de fonctionnement entre l’urgence et la Relance ?

**C. Caractéristiques et fonctionnement de l'équipe**

1. Pouvez-vous nous décrire brièvement la ***structure et le fonctionnement de l'équipe de la Relance*** ?
   1. Nombre et types de ***professionnels*** à l’équipe de Relance ?
   2. Mécanismes de ***supervision et formation continue*** ?
   3. ***Tâche*** des ***intervenants et des gestionnaires*** ?
   4. Quelles sont les principales ***compétences et aptitudes*** ainsi qu’***expériences*** nécessaires pour être de l’équipe de la Relance ?
   5. Principales ***forces et faibles*** de l’équipe de la Relance ?
2. Pouvez-vous nous décrire les ***services offerts aux patients*** ?
   1. ***Premier contact*** avec le patient ?
   2. ***Nombre et durée*** (ex.: x nbr de visites, de x nbr de minutes, sur x nombres de jours/semaines) des visites en moyenne (ainsi que les minimums et maximums) ?
   3. ***Types d’interventions*** de soutien offertes ?
   4. ***Types*** de ***référence*** effectués ? et ***avec qui*** principalement ?
   5. ***Défis*** de l’***intervention*** et de la ***référence*** ?
   6. ***Suivi à la fin de la Relance*** afin de voir si le patient est OK ou s’il a bien été transféré à un autre service ou ressource de la communauté ?
   7. ***Recommandations*** pour améliorer la prise en charge des patients après leur ***sortie de la Relance***?
3. Pouvez-vous nous décrire les ***liens de la Relance avec les autres services du Douglas*** *(autres que l’urgence) et les* ***autres partenaires du réseau?***
   1. Pour le ***Douglas***, ***globalement*** avec l’ensemble des services, et particulièrement avec le ***MIR* et le MEL**** ?
   2. Globalement avec les ***autres partenaires du réseau local***, et spécifiquement avec le ***GASMA****, le ***guichet d’accès aux médecins*** ***de famille*** et le ***centre de crise*** – l’Autre Maison ?
   3. Globalement, avec les ***ressources hors du réseau territorial*** (CIUSSS*) ?
   4. ***Autres*** partenaires s’il y a lieu ?

**D. Profil visé de patients à la Relance**

1. Comment s’établit le ***premier contact entre la Relance et les patients*** référés par l’urgence ?
2. Quels sont les critères d’***inclusion et d’exclusion*** des patients visés ?
3. Pouvez-vous nous donner une estimation du ***pourcentage des patients à l’urgence qui sont référés à votre équipe*** ?
   1. Pouvez-vous nous donner une estimation du pourcentage de patients à l’urgence qui ***devraient être transférés à votre équipe*** ?
4. Pouvez-vous nous tracer des ***portraits types de patients*** qui se présentent à vos services (profils sociodémographiques et cliniques, types de besoin, patterns au niveau des services utilisés ou types de soutien nécessaire…) ?
5. Quels sont les ***profils*** de patients ***les*** ***plus difficiles*** à « prendre en charge » à la Relance?
   1. Comment interagissez-vous lorsqu’un ***patient est réfractaire*** à l’aide que vous lui proposez ?

**Abbréviations :**

CIUSSS : Centre intégré universitaire de santé et des services sociaux

GASMA : Guichet d’accès en santé mentale adulte

MEL : Module d’évaluation liason.

MIR : Module d’intervention rapide

1. Pouvez-vous nous décrire les ***principaux défis rencontrés*** par rapport aux patients de la Relance ?
2. Vous arrive-t-il de ***revoir les patients*** plus d’une fois à la Relance ? Et si oui, quel est le ***pourcentage*** de ces patients dans vos services ? Et qu’est-ce qui ***explique cette situation*** (du point de vue des services et du profil des patients) ?
3. Pouvez-vous nous tracer les ***profils types de patients*** pour lesquels votre service semble ***bien réussir*** ?
   1. Même chose, mais profils types de ***cas d’échec*** de l’intervention ?
4. Quels sont les ***principaux éléments de succès d’une intervention efficace*** afin de bien desservir les patients transférés à la Relance ?

**E. Changements depuis l’implantation de la Relance**

1. Selon vous, quels sont les ***effets positifs les plus notables*** ***ou les principales forces*** de votre intervention sur les patients ?
2. Quels sont les ***impacts*** perçus de l’implantation de la Relance sur les services d’***urgence*** (achalandage…) ***ou autres services du réseau***?
3. Quelles sont les ***améliorations*** à apporter à la Relance en lien avec son environnement ?
4. Avez-vous d’***autres recommandations*** à formuler pour améliorer la Relance ?
   1. Quant à l’optimisation de la ***détection***, de l’***évaluation***, de la ***référence*** et du ***traitement subséquent*** des patients ?
   2. Quant aux ***profils des patients*** traités et référés ?
   3. Quant au ***fonctionnement de l’équipe***, ses relations avec l’***urgence***, l’***hôpital***, et ses ***partenaires*** ?
5. Quelles ***autres interventions ou innovations*** principalement devrait-on déployer ou consolider afin d’***améliorer les services*** aux patients ayant des troubles mentaux, la réponse à leurs besoins et l’urgence ?
6. Avez-vous d’***autres commentaires*** à formuler en lien avec la Relance, l’urgence ou l’amélioration des services aux patients (troubles mentaux et dépendance) d’une façon globale ?

***Nous vous remercions chaleureusement pour votre participation à notre projet; votre collaboration est précieuse et contribuera à nos recommandations visant à mieux répondre aux besoins des patients ayant des troubles mentaux et de dépendance !!***

**Guide d’entrevue: Centre de crise L’autre Maison**

**A. Présentation des personnes présentes**

1. Présentation et tour de table: nom & description du poste occupé

**B. Contexte d’implantation du partenariat entre l’urgence et l’Autre Maison**

1. Pouvez-vous nous présenter le ***contexte d’implantation*** du partenariat entre l’urgence et l’Autre Maison ?
   1. ***Année d’implantation*** ?
   2. Ce qui ***justifie la création*** de ce partenariat entre l’urgence et l’Autre Maison ?
   3. ***Évolution/transformation*** au cours des mois/années ?
   4. ***Difficultés d’implantation*** rencontrées et ***forces*** du processus d’implantation de ce partenariat ?
   5. ***Formalisation du processus*** s’il y a lieu ? ***Procédures*** de formalisation mises en place ? Ce qui est ici à ***améliorer*** ?
2. Comment s’effectuent les liens entre l’***urgence et l’Autre Maison*** ?
   1. Comment est ***identifié un patient*** ***à l’urgence*** pour référence à l’Autre Maison ?
   2. Comment se fait le ***processus de transfert*** du patient à l’Autre Maison ? ou la coordination entre l’Autre Maison, l’urgence et le patient ?
   3. Commentez les ***conditions de succès*** ou les ***difficultés*** des relations et modes de fonctionnement entre l’urgence et l’Autre Maison ?
   4. Au cours du processus d’implantation du partenariat, comment les relations avec les intervenants de l’urgence et de l’Autre Maison se sont-elles ***modifiées*** ?
   5. Quelles seraient les ***recommandations pour un succès optimal*** des relations et procédures de fonctionnement entre l’urgence et l’Autre Maison ?

**C. Caractéristiques et fonctionnement de l'équipe**

1. Pouvez-vous nous décrire brièvement ce ***qu’est l’Autre Maison*** ?
   1. ***Année de*** ***fondation***, ***nombre de professionnels*** équivalent temps plein (budget global), professions des intervenants à l’Autre Maison ?
   2. ***Types de services*** offerts et ***types de patients*** desservis ?
   3. ***Forces et Faiblesses*** de l’Autre Maison ?
   4. ***Historique global*** du ***partenariat*** avec le ***Douglas*** ?
2. Pouvez-vous nous décrire brièvement la ***structure et le fonctionnement du partenariat entre l’urgence du Douglas et l’Autre Maison*** ?
   1. Nombre et types de ***professionnels*** à l’équipe de l’Autre Maison pour ce partenariat ?
   2. Mécanismes de ***supervision et formation continue*** pour ce partenariat s’il y a lieu ?
   3. ***Tâches*** des ***intervenants et des gestionnaires*** pour ce partenariat ?
   4. Pensez-vous que votre intervention est adaptée aux services d’urgence du Douglas ? Commenter votre réponse.
   5. Quelles sont les principales ***compétences et aptitudes*** ainsi qu’***expériences*** nécessaires pour être de ce partenariat à l’équipe de l’Autre Maison ?
   6. Principales ***forces et faibles*** de l’équipe de l’Autre Maison pour ce partenariat ?
3. Pouvez-vous nous décrire les ***services offerts aux patients*** pour ce partenariat entre l’urgence du Douglas et l’Autre Maison ?
   1. ***Premier contact*** avec le patient ?
   2. ***Nombre et durée*** du séjour en moyenne à l’Autre Maison (ainsi que les minimums et maximums) ?
   3. ***Interventions*** de soutien offertes pendant le séjour suite au transfert de l’urgence du Douglas ?
   4. ***Type de suivi effectué*** à la suite du séjour ayant fait l’objet du transfert de l’urgence s’il y a lieu ? Processus ici standardisé ? Nombre de cas faisant l’objet de tel suivi après leur séjour suite au transfert par l’urgence ? Impact de ce suivi sur les patients ? Condition optimale d’un tel suivi ?
   5. ***Types*** de ***référence*** effectués s’il y a lieu après le séjour suite au transfert de l’urgence ? et ***avec qui*** principalement ?
   6. ***Défis*** de l’***intervention***, de la ***référence*** ou du **suivi** s’il y a lieu ?
   7. ***Recommandations*** pour améliorer la prise en charge des patients lors de leur séjour ou après leur ***séjour à l’Autre Maison***?
4. Pouvez-vous nous décrire les ***liens de l’Autre Maison avec les autres services du Douglas*** *(autres que l’urgence) et les* ***autres partenaires du réseau ?***
   1. Pour le ***Douglas***, ***globalement*** avec l’ensemble des services ?
   2. Globalement avec les ***autres partenaires du réseau local***, et spécifiquement avec le ***GASMA****, le ***guichet d’accès aux médecins*** ***de famille*** et autres ***organismes communautaires*** ?
   3. Globalement, avec les ***ressources hors du réseau territorial*** (CIUSSS) ?
   4. ***Autres*** partenaires s’il y a lieu ?

**D. Profil visé de patients pour les services offerts à l’Autre Maison dans le cadre du partenariat avec l’urgence**

1. Comment s’établit le ***premier contact entre l’Autre Maison et les patients*** transférés par l’urgence ?

**Abbréviations :**

CIUSSS : Centre intégré universitaire de santé et des services sociaux

GASMA : Guichet d’accès en santé mentale adulte

1. Quels sont les critères d’***inclusion et d’exclusion*** des patients visés par votre partenariat avec l’urgence du Douglas (profils différentiels des « patients en crises »/ versus « cas d’urgence ») ?
2. Pouvez-vous nous donner une estimation du ***pourcentage des patients à l’urgence qui sont référés à votre équipe*** ?
   1. Pouvez-vous nous donner une estimation du pourcentage des patients à l’urgence qui ***devraient être transférés*** à votre équipe ?
   2. Ces patients transférés par l’urgence du Douglas représentent ***quel pourcentage du nombre de patients*** vus ou séjournant à l’Autre Maison ?
   3. Quel est le ***nombre de lits réservés*** chez vous pour votre partenariat avec l’urgence du Douglas ?
   4. ***Combien de patients* t**ransférés de l’urgence du Douglas à l’Autre Maison ***séjournent chez vous par année***, patients transférés à l’Autre Maison de l’urgence du Douglas ?
3. Pouvez-vous nous tracer des ***portraits types de patients*** qui sont transférés à vos services dans le cadre du partenariat avec l’urgence du Douglas (profils sociodémographiques et cliniques, types de besoin, patterns au niveau de leur utilisation de services ou ceux nécessaires…) ?
4. Quels sont les ***profils*** de patients ***les*** ***plus difficiles*** à « prendre en charge » à l’Autre Maison dans le cadre du partenariat avec l’urgence du Douglas ?
   1. Comment interagissez-vous lorsqu’un ***patient est réfractaire*** à l’aide que vous lui proposez ?
5. Pouvez-vous nous décrire les ***principaux défis rencontrés*** par rapport aux patients transférés à l’Autre Maison dans le cadre de ce partenariat avec l’urgence du Douglas ?
6. Vous arrive-t-il de ***revoir les patients*** plus d’une fois à l’Autre Maison dans le cadre de ce partenariat avec l’urgence du Douglas ? Et si oui, quel est le ***pourcentage*** de ces patients dans vos services ? Et qu’est-ce qui ***explique cette situation*** (du point de vue des services et du profil des patients) ?
7. Pouvez-vous nous tracer les ***profils types de patients*** pour lesquels votre service en lien avec l’urgence du Douglas semble ***bien réussir*** ?
   1. Même chose, mais profils types de ***cas d’échec*** de l’intervention ?
8. Quels sont les ***principaux éléments de succès d’une intervention efficace*** afin de bien desservir les patients transférés à l’Autre Maison dans le cadre du partenariat avec l’urgence du Douglas ?

**E. Changements depuis l’implantation du programme: urgence avec l’Autre Maison**

1. Selon vous, quels sont les ***effets positifs les plus notables*** ***ou les principales forces*** de votre intervention sur les patients dans le cadre du partenariat développé avec l’urgence ?
2. Quels sont les ***impacts*** perçus de l’implantation du programme développé avec l’Autre Maison sur les services d’***urgence*** (achalandage…) ***ou autres services du réseau***?
3. Quelles sont les ***améliorations*** à apporter à ce partenariat entre l’Autre Maison et l’urgence du Douglas en lien avec son environnement ?
4. Avez-vous d’***autres recommandations*** à formuler pour améliorer ce programme de l’Autre Maison ?
   1. Quant à l’optimisation de la ***détection***, de l’***évaluation***, de la ***référence*** et du ***traitement subséquent*** des patients ?
   2. Quant aux ***profits des patients*** traités et référés ?
   3. Quant au ***fonctionnement de l’équipe*** pour ce programme à l’Autre Maison, ses relations avec l’***urgence***, l’***hôpital***, et ses ***partenaires*** ?
5. Quelles ***autres interventions ou innovations*** principalement devrait-on déployer ou consolider afin d’***améliorer les services*** aux patients ayant des troubles mentaux, la réponse à leurs besoins et l’urgence ?
6. Avez-vous d’***autres commentaires*** à formuler en lien avec ce programme de l’Autre Maison, l’urgence ou l’amélioration des services aux patients (troubles mentaux et dépendance) d’une façon globale ?

***Nous vous remercions chaleureusement pour votre participation à notre projet; votre collaboration est précieuse et contribuera à nos recommandations visant à mieux répondre aux besoins des patients ayant des troubles mentaux et de dépendance !!***

**Guide d’entrevue: Ami-Québec**

**A. Présentation des personnes présentes**

1. Présentation et tour de table: nom & description du poste occupé

**B. Contexte d’implantation du programme de pairs-aidants à l’urgence du Douglas en collaboration avec Ami-Québec**

1. Pouvez-vous nous présenter le ***contexte d’implantation*** du programme de pairs-aidants à l’urgence du Douglas d’Ami-Québec ?
   1. ***Année d’implantation*** ?
   2. Ce qui ***justifie la création*** de ce programme d’Ami-Québec ?
   3. ***Évolution/transformation*** au cours des mois/années ?
   4. ***Difficultés d’implantation*** rencontrées et ***forces*** du processus d’implantation ?
   5. ***Formalisation du processus*** s’il y a lieu ? ***Procédures*** de formalisation mises en place ? Ce qui est ici à ***améliorer*** ?
   6. Pouvez-vous nous expliquer la ***logique d’intégration de ce programme*** de pairs-aidants à l’urgence du Douglas au sein des autres services offerts par Ami-Québec ?
      1. Tracer-nous un ***bref portrait des services globalement d’Ami-Québec*** et de l’importance de son rôle ?
      2. Est-ce que ce service est développé dans d’***autres urgences de Montréal ou du Québec***, et ***pourquoi à l’urgence du Douglas*** ?
2. Comment s’effectue les liens entre l’***urgence et Ami-Québec*** ?
   1. Comment est ***identifié un proche-aidant d’un patient*** ***à l’urgence*** pour référence à Ami-Québec ?
   2. Comment se fait le ***processus de référence*** du proche-aidant entre les intervenants de l’urgence et Ami-Québec ?
   3. Commentez les ***conditions de succès*** ou les ***difficultés*** des relations et modes de fonctionnement entre l’urgence et Ami-Québec ?
   4. Au cours du processus d’implantation du programme de pairs-aidants à l’urgence du Douglas d’Ami-Québec, comment les relations avec les intervenants de l’urgence et d’Ami-Québec se sont-elles ***modifiées*** ?
   5. Quelles seraient les ***recommandations pour un succès optimal*** des relations et procédures de fonctionnement entre l’urgence et Ami-Québec ?

**C. Caractéristiques et fonctionnement de l'équipe**

1. Pouvez-vous nous décrire brièvement la ***structure et le fonctionnement*** du programme de pairs-aidants à l’urgence du Douglas d’Ami-Québec ?
   1. ***Nombre et profils « d’intervenants »*** dans ce programme ?
   2. Mécanismes de ***supervision et formation continue*** s’il y a lieu ?
   3. ***Tâches*** des « ***intervenants » et des gestionnaires*** d’Ami-Québec ?
   4. Quelles sont les principales ***compétences et aptitudes*** ainsi qu’***expériences*** nécessaires pour être un « intervenant » de ce programme d’Ami-Québec ?
   5. Principales ***forces et faibles*** de ce programme d’Ami-Québec ?
2. Pouvez-vous nous décrire les ***services offerts aux proches-aidants*** dans le cadre de ce programme à l’urgence du Douglas d’Amis-Québec ?
   1. ***Premier contact*** avec le proche-aidant ?
   2. ***Nombre d’activités de soutien, durée*** (année, mois) et ***intensité*** (aux deux semaines, aux mois, aux trois mois…) du soutien aux proches-aidants en moyenne (ainsi que les minimums et maximums), offert suite à la référence à l’urgence du Douglas ?
   3. ***Interventions*** de soutien offertes ou ***types de soutien*** ?
   4. ***Types*** de ***référence*** effectués à d’autres ressources pour les proches-aidants s’il y a lieu ? et ***avec qui*** principalement ?
   5. ***Défis*** du ***soutien*** et de la ***référence*** ?
   6. ***Recommandations*** pour améliorer le soutien des proches dans ce programme à l’urgence du Douglas d’***Ami-Québec***?
3. Pouvez-vous nous décrire les ***liens d’Ami-Québec avec les autres services du Douglas*** *(autres que l’urgence) et les* ***autres partenaires*** du réseau s’il y a lieu ?
   1. Pour le ***Douglas***, ***globalement*** – ensemble de ses services ?
   2. Globalement avec les ***autres partenaires du réseau de la santé mentale*** ?
   3. ***Autres*** partenaires s’il y a lieu ?

**D. Profil visé de proches-aidants pour ce programme à l’urgence du Douglas d’Ami-Québec**

1. Comment s’établit le ***premier contact entre Ami-Québec et les proches-aidants*** référés par l’urgence ?
2. Quels sont les critères d’***inclusion et d’exclusion*** des proches-aidants visés ?
3. Pouvez-vous nous donner une estimation du ***pourcentage des proches-aidants à l’urgence qui sont référés à votre équipe*** ?
   1. Pouvez-vous nous donner une estimation du pourcentage de proches-aidants à l’urgence qui ***devraient être transférés*** dans votre équipe ?
   2. D’après-vous ***combien de patients viennent à l’urgence du Douglas accompagnés de leurs proches-aidants*** ? Ce programme cible ainsi environ ***combien de proches-aidants de patients venant à l’urgence*** ?
4. Pouvez-vous nous tracer des ***portraits types de pairs-aidants*** qui se présentent à vos services (profils sociodémographiques et cliniques, types de besoin, patterns au niveau du soutien qu’ils ont déjà ou qu’ils désirent…) ?
5. Quels sont les ***profils*** de pairs-aidants ***les*** ***plus difficiles*** à « prendre en charge » dans ce programme à l’urgence du Douglas d’Ami-Québec?
   1. Comment interagissez-vous lorsqu’un ***proche-aidant est réfractaire*** à l’aide ou aux types d’aide que vous lui proposez ?
6. Pouvez-vous nous décrire les ***principaux défis rencontrés*** par rapport aux proches-aidants dans ce programme à l’urgence du Douglas d’Ami-Québec ?
7. Pouvez-vous nous tracer les ***profils types de proches-aidants*** pour lesquels votre service semble ***bien réussir*** ?
   1. Même chose, mais profils types de ***cas d’échec*** de l’intervention ?
8. Quels sont les ***principaux éléments de succès d’une intervention efficace*** afin de bien desservir les proches-aidants transférés par l’urgence à ce programme d’Ami-Québec ?

**E. Changements depuis l’implantation de ce programme à l’urgence du Douglas d’Ami-Québec**

1. Selon vous, quels sont les ***effets positifs les plus notables*** ***ou les principales forces*** de votre programme sur les proches-aidants et les patients ?
2. Quels sont les ***impacts*** perçus de l’implantation de ce programme d’Ami-Québec sur les services d’***urgence*** du Douglas ***ou autres services du réseau***?
3. Avez-vous d’***autres recommandations*** à formuler pour améliorer ce programme à l’urgence du Douglas d’Ami-Québec ?
   1. Quant à l’optimisation de la ***détection***, de l’***évaluation***, de ***soutien*** et les ***références nécessaires subséquentes*** pour les proches-aidants ?
   2. Quant aux ***profits des proches-aidants*** soutenus et référés ?
   3. Quant au ***fonctionnement de votre « équipe »***, ses relations avec l’***urgence***, l’***hôpital***, et ses ***partenaires*** le cas échéant ?
4. Quelles ***autres interventions ou innovations*** principalement devrait-on déployer ou consolider afin d’***améliorer les services*** aux proches-aidants et aux patients à l’urgence et ailleurs ?
5. Avez-vous d’***autres commentaires*** à formuler en lien avec Ami-Québec, l’urgence ou l’amélioration des services aux patients ?

***Nous vous remercions chaleureusement pour votre participation à notre projet; votre collaboration est précieuse et contribuera à nos recommandations visant à mieux répondre aux besoins des patients ayant des troubles mentaux et de dépendance !!***
